# Mechanisms underlying the antiproliferative effects of a series of quinoxaline-derived chalcones

**DOI:** 10.1038/s41598-017-16199-3

**Published:** 2017-11-20

**Authors:** Tânia R. Mielcke, Thaís C. Muradás, Eduardo C. Filippi-Chiela, Maria Eduarda A. Amaral, Luiza W. Kist, Maurício R. Bogo, Alessandra Mascarello, Patrícia D. Neuenfeldt, Ricardo J. Nunes, Maria M. Campos

**Affiliations:** 1 Postgraduate Program in Medicine and Health Sciences, PUCRS, Porto Alegre, RS Brazil; 2 Institute of Toxicology and Pharmacology, PUCRS, Porto Alegre, RS Brazil; 3 Postgraduate Program in Hepatology and Gastroenterology, UFRGS, Porto Alegre, RS Brazil; 4 Postgraduate Program in Cellular and Molecular Biology, PUCRS, Porto Alegre, RS Brazil; 5 Laboratory of Genomics and Molecular Biology, PUCRS, Porto Alegre, RS Brazil; 6 Department of Chemistry, UFSC, Florianópolis, SC Brazil; 7 School of Dentistry, PUCRS, Porto Alegre, RS Brazil

## Abstract

The present study aimed to characterize the effects of quinoxaline-derived chalcones, designed on the basis of the selective PI3Kγ inhibitor AS605240, in oral cancer cells. Three lead compounds, namely N9, N17 and N23, were selected from a series of 20 quinoxaline-derived chalcones, based on an initial screening using human and rat squamous cell carcinoma lineages, representing compounds with at least one methoxy radical at the A-ring. The selected chalcones, mainly N9 and N17, displayed marked antiproliferative effects, via apoptosis and autophagy induction, with an increase of sub-G1 population and Akt inhibition. The three chalcones displayed marked *in vitro* antitumor effects in different protocols with standard chemotherapy drugs, with acceptable toxicity on normal cells. There was no growth retrieval, after exposure to chalcone N9 alone, in a long-term assay to determine the cumulative population doubling (CPD) of human oral cancer cells. A PCR array evaluating 168 genes related to cancer and inflammation, demonstrated striking actions for N9, which altered the expression of 74 genes. Altogether, our results point out quinoxalinic chalcones, mainly N9, as potential strategies for oral cancer treatment.

## Introduction

Head and neck cancers (HNSCC) encompass tumor types arising from many sites in the upper aerodigestive tract. More than 90% of cases are squamous cell carcinomas, which occur most frequently in the oral cavity, oropharynx and larynx^1^. The oral squamous cell carcinoma (OSCC) is the most common type^1^. Regardless of the vast number of studies and the development of new and less toxic treatment regimens, in addition to the advances in diagnosis tools, the survival rates have not significantly changed over the past decades^2^. The five-year survival rate of patients with OSCC remains below 50%; besides, around 70% of advanced-stage cases are incurable^3^. For these reasons, OSCC remains a significant worldwide disease burden^4^. The poor outcome can partly be related to the development of resistance to radiation and chemotherapy, with loco-regional and distant failures^2^, or the occurrence of a second primary tumor^5^. Therefore, novel and effective therapeutic options for treating these tumors are needed.

Molecules based on natural products have a relevant role in oncology drug discovery, and several natural product-derived compounds present beneficial effects when combined with classical chemotherapeutic drugs. Chalcones (1,3-diphenyl-2-propen-1-one) are a group of natural precursors of flavonoid biosynthesis in high plants^6^, presenting a broad spectrum of biological activities, such as anti-cancer, antioxidant, anti-inflammatory, antibacterial and antimalarial^6,7^. Chemically, these compounds are open-chained molecules composed of two aromatic rings joined by three unsaturated α, β carbons and one carbonyl group^8^. The simple structure and the easy process for obtaining these compounds make them interesting for structure-activity relationship (SAR) studies^7^. Numerous substituents were linked to the chalcone scaffold, and different series of effective synthetic analogs with therapeutic potential for many cancer types were obtained. These structural modifications produced a great variety of compounds with different mechanisms of action^9^. Previous data showed that compounds with a quinoxaline ring in their structure have the ability to inhibit the angiogenic process^10^, and to induce caspase-dependent apoptotic cell death^11^. Some additional antiproliferative mechanisms also support the notion that such compounds might be potential candidates for cancer treatment^10^. Previous studies from our group and co-workers demonstrated that different chalcones derived from quinoxaline and based on the selective PI3Kγ inhibitor AS605240, showed a great capacity to inhibit cell proliferation and to reduce the viability of glioma cell lines^12,13^. With this in mind, the present study aimed to evaluate the action of twenty quinoxaline-derived chalcones in different OSCC cell lineages. Attempts have been made to characterize the anti-cancer activity of the most effective compounds, presenting at least one methoxy radical on the A-ring, focusing on the mechanisms of action underlying its effects in OSCC cells, alone or in combination with classical anti-cancer drugs in clinically relevant treatment protocols.

## Results and Discussion

The first set of experiments was conducted to select the quinoxaline-derived chalcones with the greatest cytotoxic effect on OSCC lines, based on the reduction of cell viability assessed through the MTT assay. Human HN30 (Supplementary Figures S1 to S4) and rat SCC-158 cell lines (Supplementary Figures S5 to S8) were treated with 20 different compounds at concentrations varying from 0.29 µM to 38.42 µM, for 24, 48 or 72 h. Data obtained with the 20 compounds were presented separately, according to the number of methoxy radicals on the A-ring, as monomethoxylated, di-methoxylated, tri-methoxylated and non-methoxylated (Supplementary Figures S1–S8). The antitumor effects of these compounds, on both cell strains, revealed a concentration- and time-dependent profile.

Several previous studies demonstrated that the biological activities of chalcones are related to the chemical structure, especially when substituents are added to both aromatic rings of the fundamental nucleus^9^. Among the twenty compounds evaluated in this work, seven presented maximal percentages of inhibition (Imax) around 50% after 48 h, according to assessment of both cell lines. The IC_50_ values were greater than 30 μM for most tested compounds. Interestingly, the chalcones with higher cytotoxic potential display at least one methoxy group at the phenyl A-ring of its structure (Table 1). Therefore, in sequence we selected a monomethoxylated (N23; 3′-OCH3), a dimethoxylated (N9; 2′,5′-diOCH3) and a trimethoxylated chalcone (N17; 2′,4′,5′-triOCH3), to evaluate whether the number of methoxy radicals at the A-ring might influence the anti-tumor effects of the compounds.

**Table 1:** Effects of quinoxaline-derived chalcones on the viability of human and rat oral squamous cell carcinoma (OSCC) cell lines.

Chalcones		OSCC Cell Line	
		Human HN30 Imax (%)^a^	Rat SCC-158 Imax (%)^a^
N9	2′,5′-di OCH_3_	35.65 ± 12.29%	59.78** ± **5.40%
N15	3′,4′,5′-tri OCH_3_	43.65 ± 13.60%	50.65 ± 6.80%
N16	3′-OCH3, 4′-OH	41.07 ± 12.12%	29.23 ± 6.59%
N17	2′,4′,5′-tri OCH_3_	52.90 ± 11.40%	51.27 ± 10.8%
N18	3′,5′-di OCH_3_	48.06 ± 10.30%	43.27 ± 10.75%
N20	3′,5′-di OCH_3_, 4′-OH	54.30 ± 12.70%	31.06 ± 6.87%
N23	3′-OCH_3_	42.09 ± 5.21%	49.56 ± 4.47%

^a^The maximal percentages of inhibition were calculated for: N9 (15.61 μM); N15 (14.27 μM); N16 (16.32 μM); N17 (14.27 μM); N18 (15.61 μM); N20 (14.87 μM); N23 (17.22 μM). All the compounds were incubated for 48 h.

Winter *et al*. evaluated some of these quinoxaline-derived chalcones on breast cancer cell lines, and proposed that the contribution of methoxy group can be related to electrostatic and steric features^14^. Many methoxylated chalcones with anticancer activities were reported by different research groups during the last years^15^. Besides the substituents on the A-ring, the quinoxaline group on B-ring might favor the cytotoxic action. This seems to rely on the electrostatic interactions of the two nitrogen heteroatoms^14^. Others substituents, including Cl, Br, NO_2_, OH, CN, or CF_3_ did not present the same inhibition activity, probably due to their hydrophilicity^14^. A similar profile for these compounds was also observed in this study, using OSCC cell lines.

As mentioned before, from the seven compounds identified in the initial screening tests, three were chosen for additional biological assays. The selected compounds were chalcone N9 (2′,5′-diOCH3), chalcone N17 (2′,4′,5′-triOCH3) and chalcone N23 (3′-OCH3). Hereafter, this study was designed to define the potential cellular targets of lead compounds, and for comparison and combination with standard chemotherapies clinically used in the treatment of OSCC.

**Figure 1:**
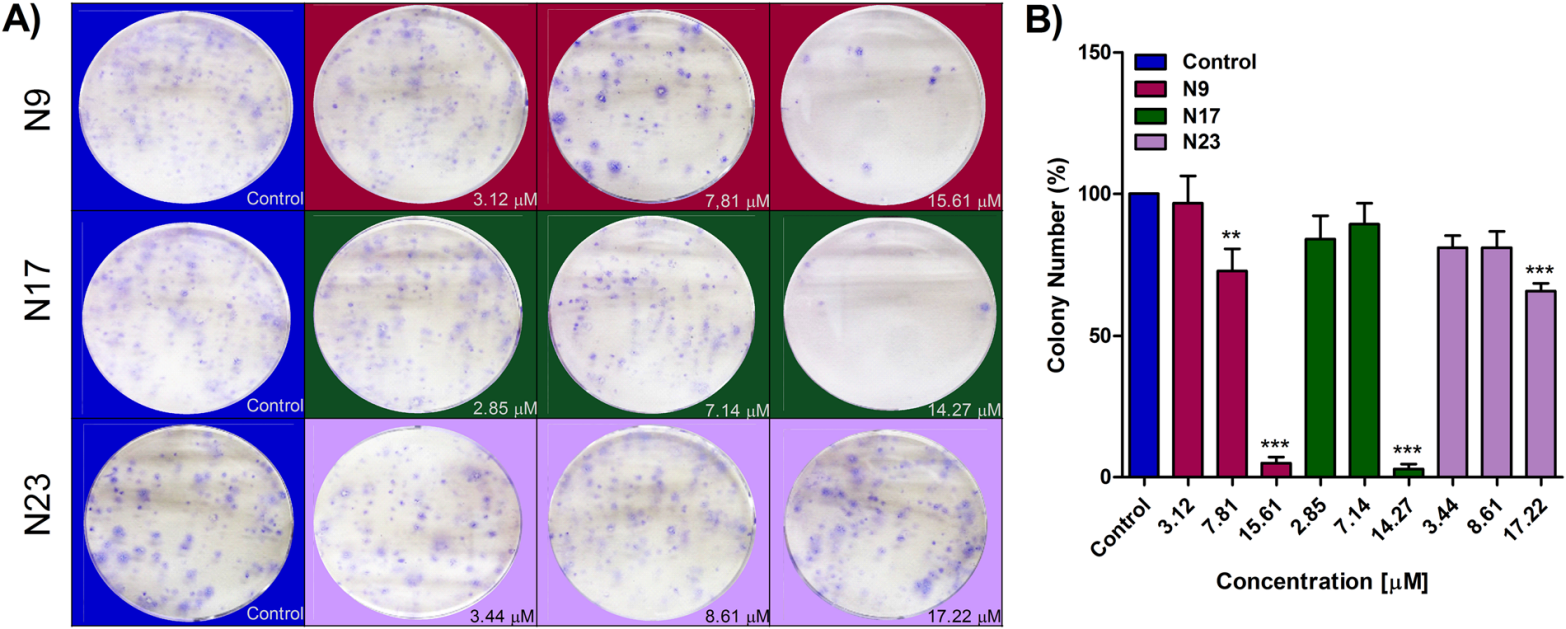
(**A**,**B**) Effects of chalcones N9, N17 and N23 in the clonogenic assay. (**A**) Representative images of the clonogenic assay; HN30 cells were treated with the chalcones N9 (red panels; 3.12 µM, 7.81 µM and 15.61 µM), N17 (green panels; 2.85 µM, 7.14 µM and 14.27 µM) and N23 (lilac panels; 3.44 µM, 8.61 µM and 17.22 µM), for 48 h. After this period, the cells were maintained in a drug-free medium for 12 days. (**B**) Bar chart demonstrating the percentage of colony numbers of HN30 cells after 12 days. Data represent the mean of 3 independent experiments, performed in duplicate, and the lines indicate the standard error mean. Data were analyzed by one-way ANOVA, followed by Dunnet’s post-test; the group A represents the negative control. Significantly different from the control group (*P < 0.05); (**P < 0.01), (***P < 0.001).

As a first approach, HN30 cells were treated with chalcones N9 (3.12 µM, 7.81 µM and 15.61 µM), N17 (2.85 µM, 7.14 µM and 14.27 µM) and N23 (3.44 µM, 8.61 µM and 17.22 µM), for 48 h, and a clonogenic assay was performed. When compared to the control, a significant decrease in colony formation was observed after treatment with chalcones N9 (15.61 µM) and N17 (14.27 µM) (Fig. 1). This data suggests that both compounds strongly reduce the formation of new cancer cell colonies after treatment, when compared to the chalcone N23.

**Figure 2:**
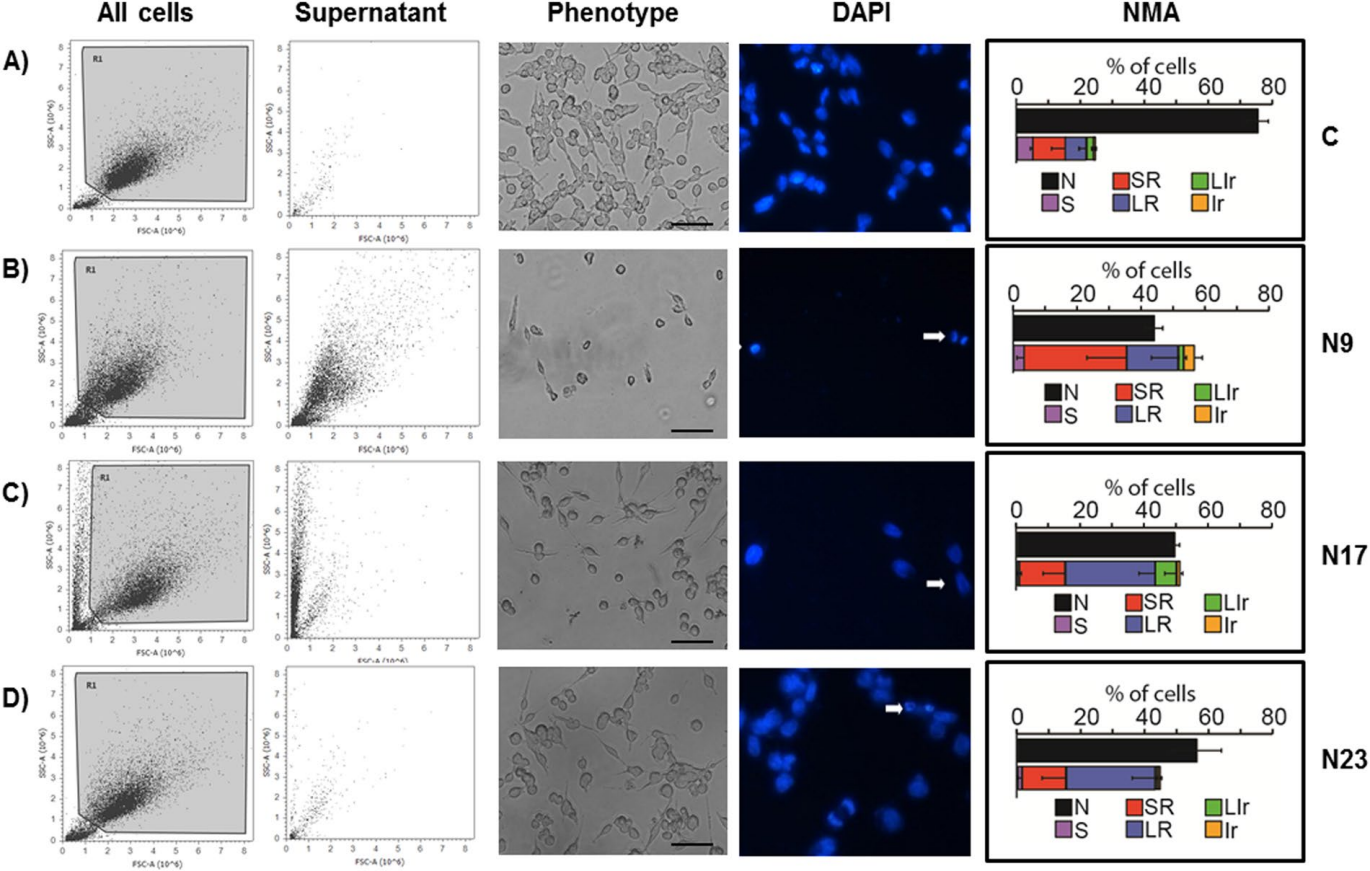
(**A**,**D**) Effects of chalcones N9, N17 and N23 on phenotypic characteristics and cell death of HN30 cells. The HN30 cells were treated with the chalcones N9 (15.61 µM), N17 (14.27 µM) and N23 (17.22 µM), for 48 h. (**A**) Control cells. (**B**) Cells treated with chalcone N9. (**C**) Cells treated with chalcone N17. (**D**) Cells treated with chalcone N23. The first and the second columns represent all cells or the supernatant of the cells analyzed by flow cytometry, respectively. The third column shows the morphology of the cells on microscopy and the fourth column the cell staining with DAPI. The white arrows represent cells in apoptosis. The last column shows the NMA analysis. N: Normal nuclei, Ir: irregular, LR: Large Regular; LIr: Large Irregular; SR: Small and Regular; S: Small.

**Figure 3:**
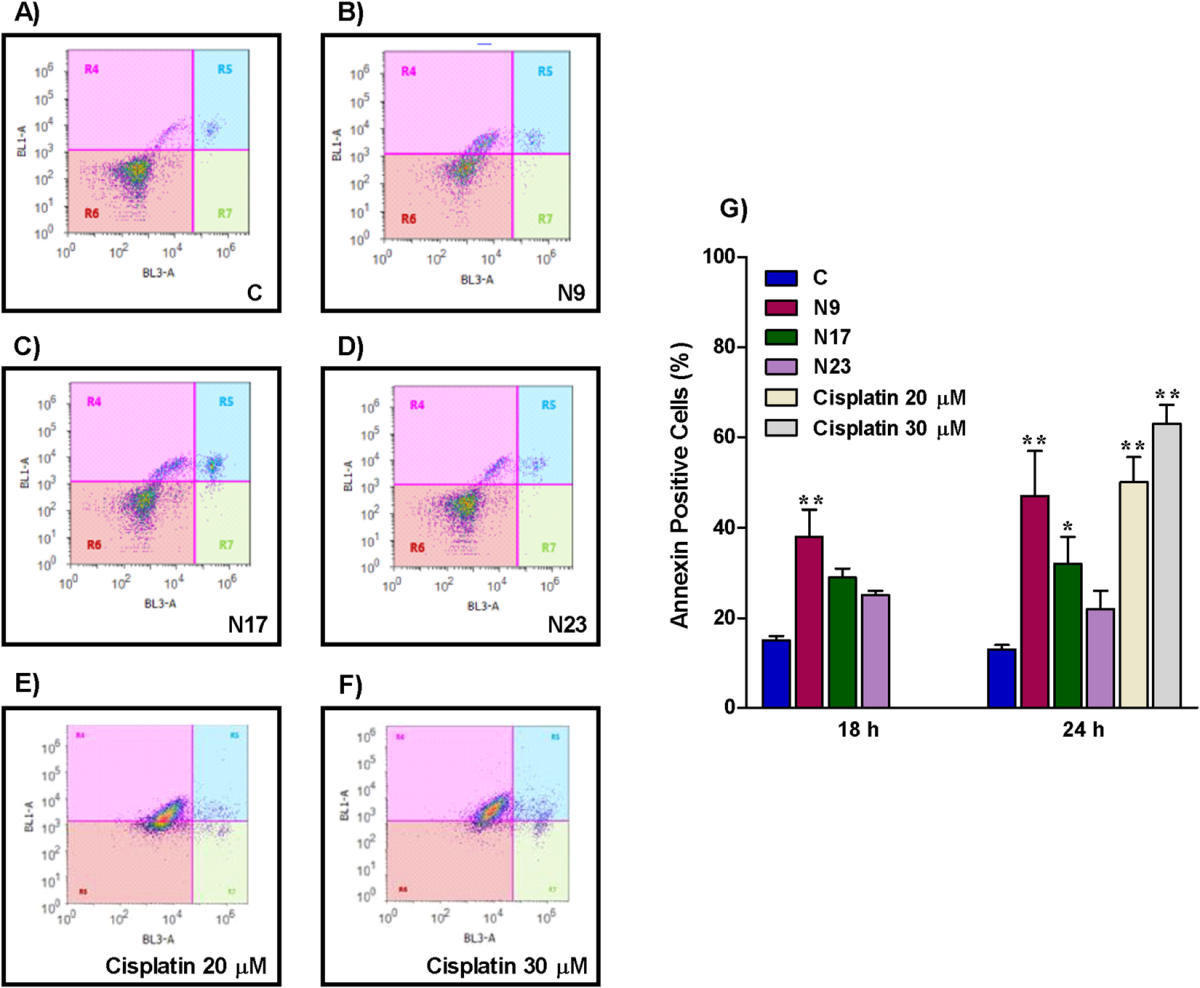
(**A**,**E**) Effects of chalcones N9, N17 and N23 on cell death of HN30 cells. (**A**,**F**) Histogram analysis of annexin V/FITC at 24 h of treatment; R4 quadrant, annexin-positive cells; R5 quadrant, annexin/ PI-positive cells; R6, unmarked cells; R7 quadrant, PI-positive cells. (**G**) Bar chart showing the percentage of HN30 cells stained with FITC after treatment with the chalcones N9 (15.61 µM), N17 (14.27 µM) and N23 (17.22 µM), for 18 and 24 h. Cisplatin (20 and 30 μM; 24 h) was used as a positive control drug. Each column represents the mean of 3 independent experiments performed in triplicate, and the lines indicate the standard error mean. Data were analyzed by two-way ANOVA, followed by Bonferroni’s post-test; the group A represents the negative control. Significantly different from the control group (*P < 0.05); (**P < 0.01).

Apoptosis is an important target for cancer treatment, and a wide number of chalcones and other natural products present pro-apoptotic effects^8,9^. Chalcones N9 and N17 caused marked phenotypic changes in HN30 cells, when compared to chalcone N23 and to the negative control. A decrease of forward scatter signal (FSC) on the flow cytometry (cell size) was observed after the treatment with N9 and N17 (Fig. 2, first column). Based on this characteristic, it is possible to infer that oral cancer cells treated with selected chalcones, especially N9 and N17, undergo apoptosis. The induction of cell death by the lead compounds was also demonstrated by assessment of the cell numbers in the supernatant (Fig. 2, second column). To further characterize the cell death process caused by the quinoxaline-related chalcones in oral cancer, the HN30 cells were treated with N9 (15.61 µM), N17 (14.27 µM) or N23 (17.22 µM), for 48 h, and then stained with DAPI. We observed differences in the nuclear morphology when comparing untreated and treated groups, especially in N9- and N17-treated cells. Morphological features, such as shrinking, irregular surface and nuclear fragmentation can be observed, as indicated by the arrows and at higher magnification, in fluorescence microscopy images (Fig. 2, fourth column), which are all indicatives of apoptotic process^16^. Similar features were seen at the light microscopy (Fig. 2, third column). Moreover, the nuclear morphometric analysis (NMA) indicates an increase in the SR (small regular) population cells, corroborating the induction of apoptosis by the lead chalcones, especially by N9 (Fig. 2, fifth column). The chalcones N17 and N23 also elicited changes of LR (large regular), which is an indicative of senescence. Complete data on NMA analysis is represented in the Supplementary Figure S9. To extend this data, we performed an annexin-PI assay to specifically assess and confirm the induction of apoptosis. Corroborating data from cellular and nuclear morphology, chalcones N9 and N17 increased the percentage of annexin-positive cells to around 50% and 40% after 24 h, respectively, whereas chalcone N23 induced apoptosis in approximately 20% of the cells. The positive control drug cisplatin (20 and 30 μM) also induced an increase in the percentage of annexin-positive cells, at the same time-point (50% to 60%) (Fig. 3). Necrosis induction was not observed with either tested chalcone (Fig. 3; R7 quadrant). Altogether, these results suggest that N9 was the most cytotoxic chalcone tested, both acutely (mainly through apoptosis induction) and at a long-term (as assessed by the clonogenic assay), followed by N17 and N23, respectively.

**Figure 4:**
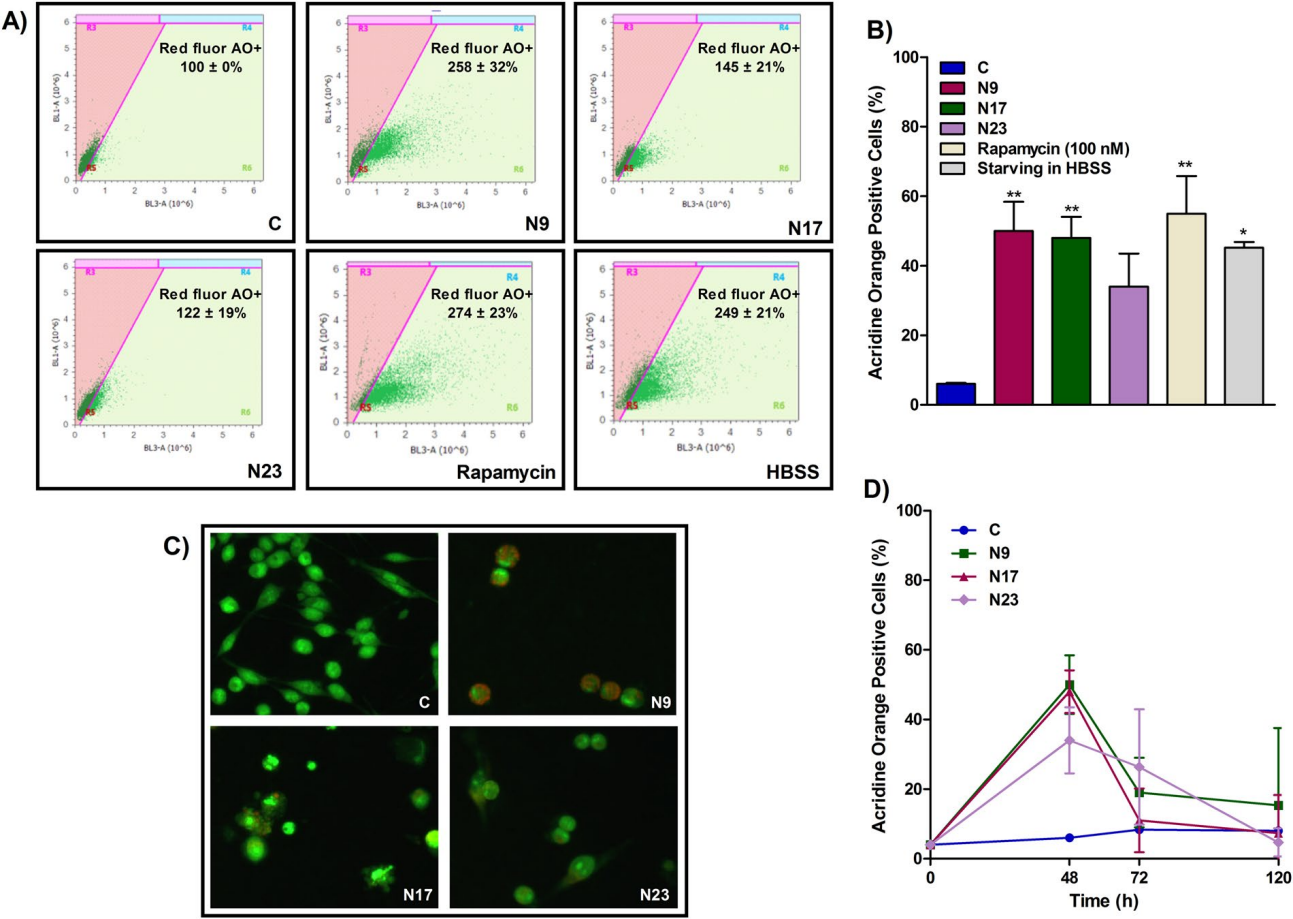
(**A**,**F**) Effects of chalcones N9, N17 and N23 on autophagy of HN30 cells. (**A**) Histogram analysis showing the intensity of red fluorescence in acridine orange (AO)-positive cells. (**B**) Bar chart showing the effects of the chalcones N9 (15.61 µM), N17 (14.27 µM) and N23 (17.22 µM), for 48 h, on the percentage of AO-positive HN30 cells. This panel also shows the effects of the positive control drug rapamycin (100 nM) or the starving in Hanks’ Balanced Salt solution (HBSS) on autophagy. (**C**) Representative fluorescence microscopy images of HN30 cells treated with N9 (15.61 µM), N17 (14.27 µM) and N23 (17.22 µM), for 48 h. (**D**) Percentage of AO-positive HN30 cells, after the 48, 72 and 120 h. The cells were treated with N9, N17 and N23, for 48 h. After this period, the cells were maintained in a drug-free medium. Data represent the mean of 3 independent experiments performed in triplicate, and the lines indicate the standard error mean. Data were analyzed by one-way ANOVA, followed by Dunnet’s post-test; the group A represents the negative control. Significantly different from the control group (*P < 0.05); (**P < 0.01).

To analyze other mechanisms that might be involved in the inhibition of OSCC proliferation and death process, we evaluated the effects of the lead quinoxaline-derived chalcones in autophagy. In cancer, autophagy is a double-edged sword, responsible for protecting or killing tumor cells. As a cytoprotective mechanism, autophagy act as an adaptive mechanism in response to stress conditions and is associated with high metabolic demands, being able to trigger the resistance of cancer cells against chemo- and radiation therapy^17^. Vessoni *et al*. proposed that this cytoprotective tool can be related to the degradation of pro-apoptotic proteins, the induction of DNA repair supplied by ATP and dNTPs, besides the regulation of cell cycle arrest and mitochondria functions^18^. Conversely, autophagy can favor tumor cell death through degradation of proteins with anti-apoptotic and/or DNA-repairing actions^18^, and the modulation of several metabolic signaling pathways, such mTOR, PI3K, MAPKs and PKC^19^. Here, we found that chalcones induced an increase in the percentage of AO-positive cells (Fig. 4B,C). N9 had the strongest pro-autophagic effect, followed by N17 and N23. It is important to observe that, despite N9 and N17 increased the percentage of AO-positive cells to similar levels, N9 increased the intensity of red fluorescence in AO-positive cells to a higher extent than N17, which suggest an increased level of autophagy in single cells (Fig. 4A). The percentage of AO-positive cells was also increased by the incubation of the positive control drug rapamycin (100 nM), or by starvation with Hanks’ Balanced Salt solution (HBSS) (Fig. 4B). In an attempt to integrate all the abovementioned results, we used Pearson’s correlation coefficients between the reduction in cell number, the percentage of apoptosis and the percentage of autophagy. The reduction in cell number strongly correlated negatively with the induction of apoptosis (ρ = −0.86); the percentage of AO-positive cells strongly correlated positively with the percentage of annexin-positive cells (ρ = 0.81); finally, we found a strong negative correlation between the reduction of cell number and the percentage of AO-positive cells (ρ = −0.98). Altogether, these results suggest that the sensitivity to apoptosis and autophagy was similar to a single stimulus (i.e. the higher the pro-apoptotic effect, the greater the pro-autophagic effect), and that these mechanisms may explain, to a higher extent, both the cytotoxicity and the resistance of OSCC cells to chalcones.

The maintenance of autophagy at a long term, even after the withdrawal of the cytotoxic treatment, can influence the cell fate, which can be clinically relevant. Since autophagy was highly induced in those cells that resisted to the treatment with the chalcones, we next assessed whether autophagy would persist after the withdrawal of the compounds^17^. For this, the HN30 cells were treated for 48 h, and then maintained in a medium free of chalcones. After 72 h and 120 h, the cells were stained with AO and analyzed by flow cytometry. Data demonstrate that the autophagy induced by quinoxalinic chalcones in OSCC cells is dependent on the presence of the compounds in the medium, since autophagy decreased after drug removal (Fig. 4D). This suggests that chalcones do not cause a permanent or sustained damage to cellular structures, in opposition to DNA damage agents, which induced an increase in autophagy even after drug removal^17^.

**Figure 5:**
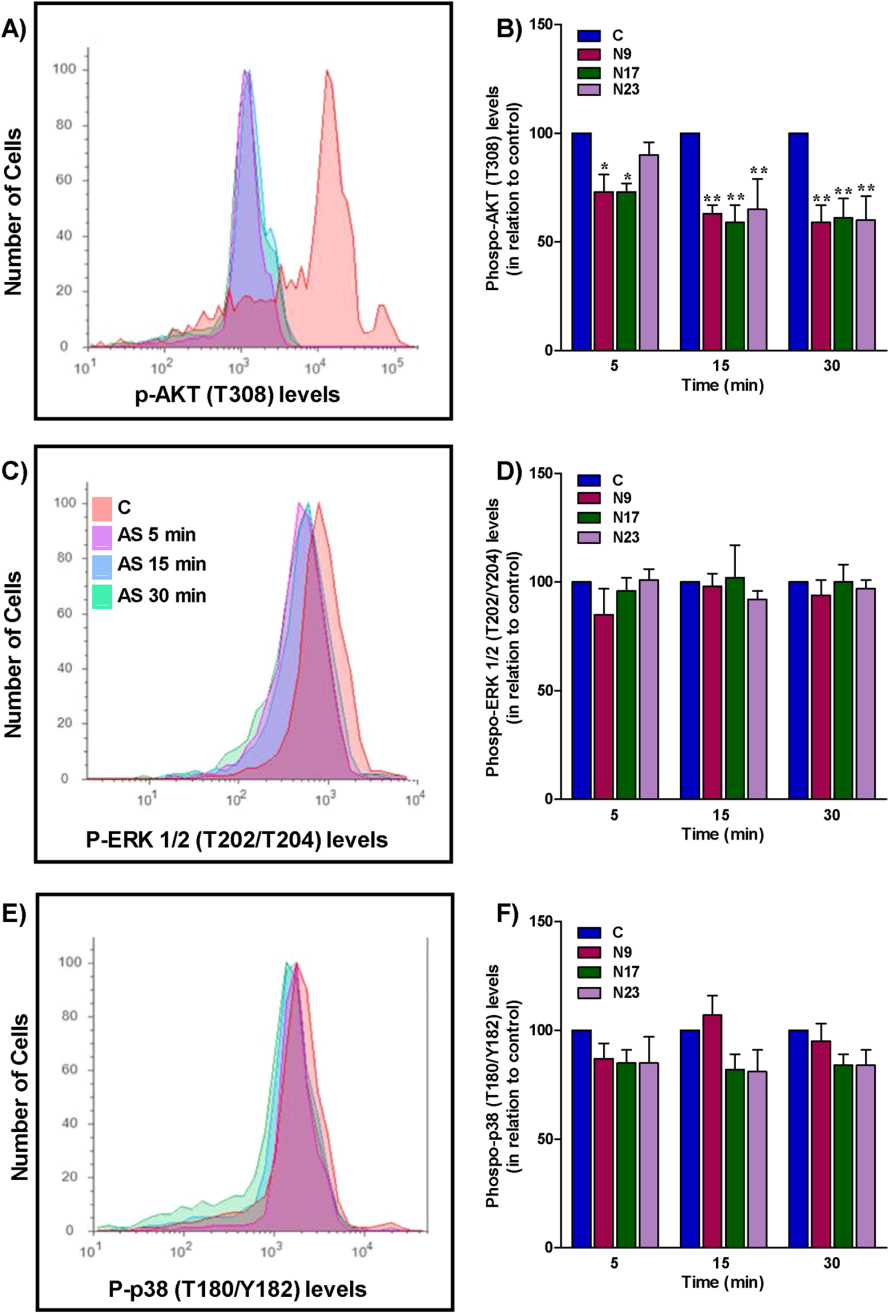
(**A**,**C**) Effects of chalcones N9, N17 and N23 on the activation of MAP kinases ERK 1/2 and p38, or the PI3K-related serine/threonine kinase AKT. HN30 cells were treated with the chalcones N9 (15.61 µM), N17 (14.27 µM) and N23 (17.22 µM), for 5, 15 or 30 min. (**A**,**B**) Histogram and bar chart showing the Akt phosphorylation. (**C**,**D**) Histogram and bar chart showing the Erk 1/2 phosphorylation. (**E**,**F**) Histogram and bar chart showing the p38 phosphorylation. The columns represent the mean of 3 independent experiments, and the lines indicate the standard error mean. Data were analyzed by two-way ANOVA, followed by Bonferroni’s post-test. Significantly different from the control group (*P < 0.05); (**P < 0.01).

To unravel the mechanisms underlying the inhibition of cell proliferation, and the induction of apoptosis and autophagy, the effects of the selected chalcones were examined on signaling proteins involved in cell survival and growth, such as phosphatidylinositol 3-kinase/protein kinase B (PI3K/Akt) and mitogen-activated protein kinases (MAPK). The signaling pathway phosphatidylinositol 3-kinase/protein kinase B/ mammalian target of rapamycin (PI3K/Akt/mTOR) is involved in the control of cell cycle, survival, proliferation, metabolism and motility, which are important hallmarks of cancer^20^. Genomic studies demonstrated that this route is frequently altered in human tumors, including head and neck cancer^21^. Compelling evidence showed PIK3CA mutations in OSCC, suggesting that this gene can be related to OSCC tumorigenesis, contributing to oncogene activation^21^. The PI3K/Akt/mTOR signaling pathway also plays a critical role in autophagy, as mTOR is a metabolic regulator of autophagy^22^. When nutrients are abundant and the cell is in homeostasis, mTOR acts to inhibit the autophagosomes and consequently, the autophagy. However, when mTOR is inhibited by cell stress or the PI3K/Akt/mTOR pathway is blocked, an increase of autophagy can be observed^22^. The first PI3K inhibitor for cancer treatment was recently approved in the USA. Idelalisib, a selective PI3Kδ inhibitor, was licensed in 2014 for the treatment of various hematologic malignancies^23^. The search for new inhibitors which can be used in the treatment of other tumors remains greatly necessary. Considering the importance of PI3K in tumorigenesis, apoptosis and autophagy, and that quinoxaline-related chalcones assessed in this study had their structure based on the selective PI3Kγ inhibitor AS605240, it is reasonable to investigate the relevance of this signaling molecule in the effects of chalcones. Thus, HN30 cells were treated with chalcones N9, N17, N23 or AS605240, for 5, 15 and 30 min, and the effect on Akt phosphorylation was analyzed by flow cytometry. The three compounds reduced the phosphorylation of Akt, mainly after 15 and 30 min of incubation (Fig. 5A,B). Akt phosphorylation was also markedly prevented by AS605240 at every tested time-point (Supplementary Figure 10A,B). These results suggest that the punctual modulation of Akt phosphorylation by these chalcones may be related to the antiproliferative, apoptotic and autophagic effects observed.

The mitogen-activated protein kinases (MAPK) are also involved in cell growth, proliferation, survival, autophagy and apoptosis. JNK, Erk 1/2 and p38 MAPK are key regulatory proteins in these processes^24^. To evaluate the effects of the quinoxaline-derived chalcones on Erk 1/2 and p38 activation, we used the same protocol described previously for Akt phosphorylation analysis. The chalcones N9, N17and N23 did not alter the status of Erk 1/2 or p38 activation in HN30 cells (Fig. 5C–F), implying that antitumor actions of the lead chalcones tested in this study are not likely related to MAPK pathway modulation. AS605240 also failed to modify Erk 1/2 and p38 MAPK phosphorylation (Supplementary Figure 10C–F).

**Figure 6:**
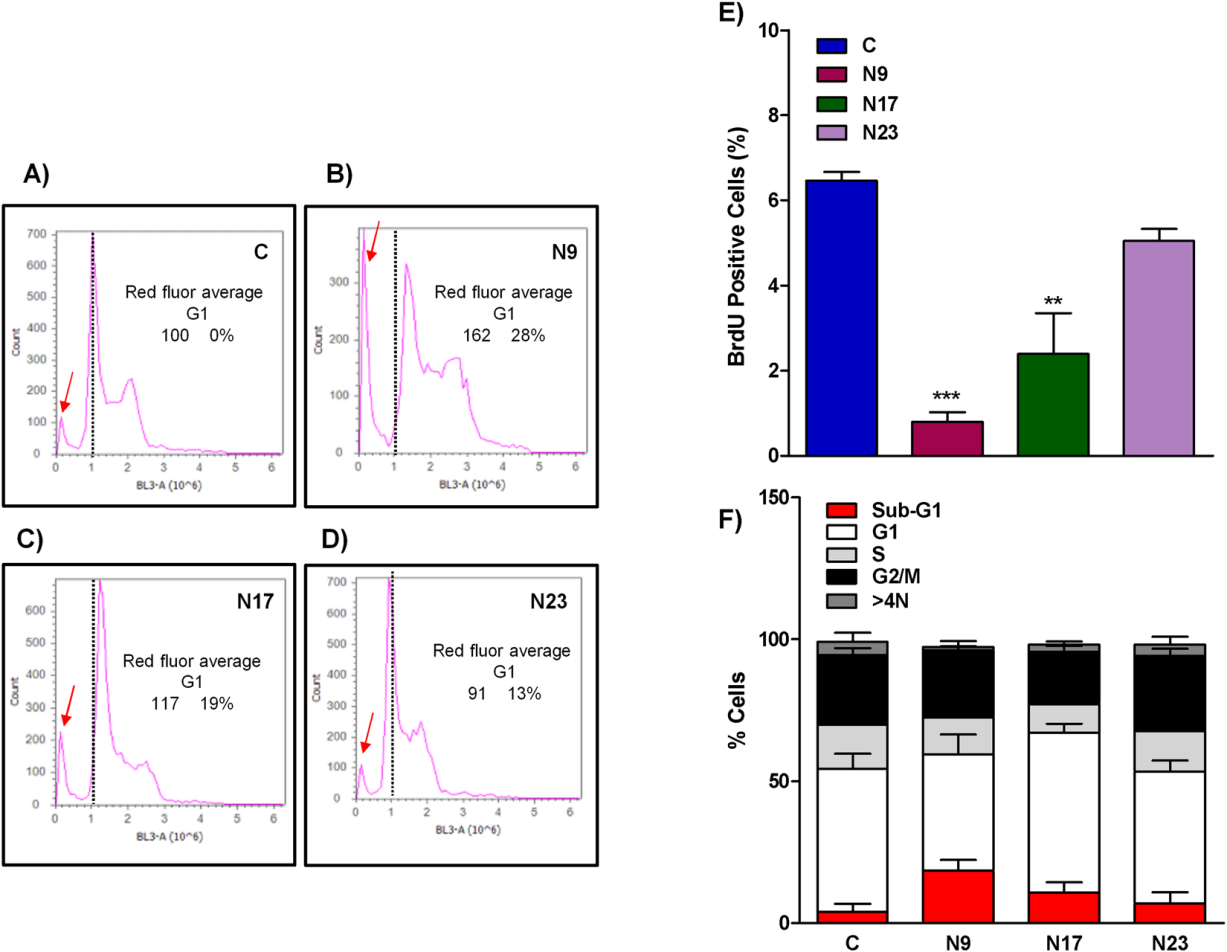
(**A**,**E)** Effects of chalcones N9, N17 and N23 on the cell cycle. (**A**,**D**) histogram analysis of Red Fluor G1-positive cells; (**E**) Bar chart showing the percentage of positive HN30 cells stained with BrdU and treated with chalcones N9 (15.61 µM), N17 (14.27 µM) and N23 (17.22 µM), for 48 h; (**F**) Bar chart showing the percentage of HN30 on sub-G1 phase (red), G1 (white), S (light grey), G2/M (black) and >4 N (dark grey). Data represent the mean of 2 independent experiments performed in duplicate, and the lines indicate the standard error mean. Data were analyzed by one-way ANOVA, followed by Dunnet’s post-test; the group A represents the negative control. Significantly different from the control group (*P < 0.05); (**P < 0.01).

An important mechanism for tumor control involves the modulation of the cell cycle. Numerous natural compounds, including chalcones, demonstrated an ability to control the cell cycle at the different phases^9^. To verify if the selected quinoxalinic chalcones interfere with the cell cycle progression of OSCC, HN30 cells were treated with chalcones N9, N17 and N23, then marked with BrdU and analyzed with flow cytometry, after 48 h. An increase in sub-G1 population was observed, especially when cells were treated with N9 and N17 (Fig. 6A–D, red arrows), corroborating with the induction of apoptosis as abovementioned (Fig. 6F). N9 and N17 also reduced the percentage of BrdU-labeled cells, suggesting that a reduction in cell proliferation, in combination with apoptosis induction, may be involved in the cytotoxicity of tested compounds (Fig. 6E). The chalcone N9 also induced an increase of sub-G1 population, according to the evaluation of C6 rat glioma cells^12^.

The treatment of oral cancer should be individualized, taking into account the stage of the lesion, anatomical location, size and tumor histology, in addition to the general conditions of the patient^1^. Besides surgery and radiotherapy, the treatment of OSCC can also comprise chemotherapy. The main chemotherapy agents used include platinum compounds, taxanes, 5-fluorouracil, methotrexate and/or cetuximab, commonly used in treatment protocols^25^. Thereupon we examined the viability of HN30 cells after treatment with the selected quinoxalinic-derived chalcones (N9, N17 and N23) in different schemes of combination with cisplatin, docetaxel and 5-fluoruracil, using subthreshold concentrations, at different time-points. The selective PI3Kγ inhibitor AS605240 was included in this analysis, as it was used as the prototype for the synthesis of the quinoxaline-related chalcones used herein. The tested chalcones or AS605240, at subthreshold concentrations, incubated alone or in combination, failed to alter the viability of HN30 cells, as evaluated in the MTT assay (Supplementary Table S1). However, the association of each chalcone with cisplatin or 5-fluorouracil resulted in marked antiproliferative effects, especially at 48 h and 72 h, with chalcone N9. Similar percentages of inhibition were observed by the combination of the three reference chemotherapy drugs.

**Figure 7:**
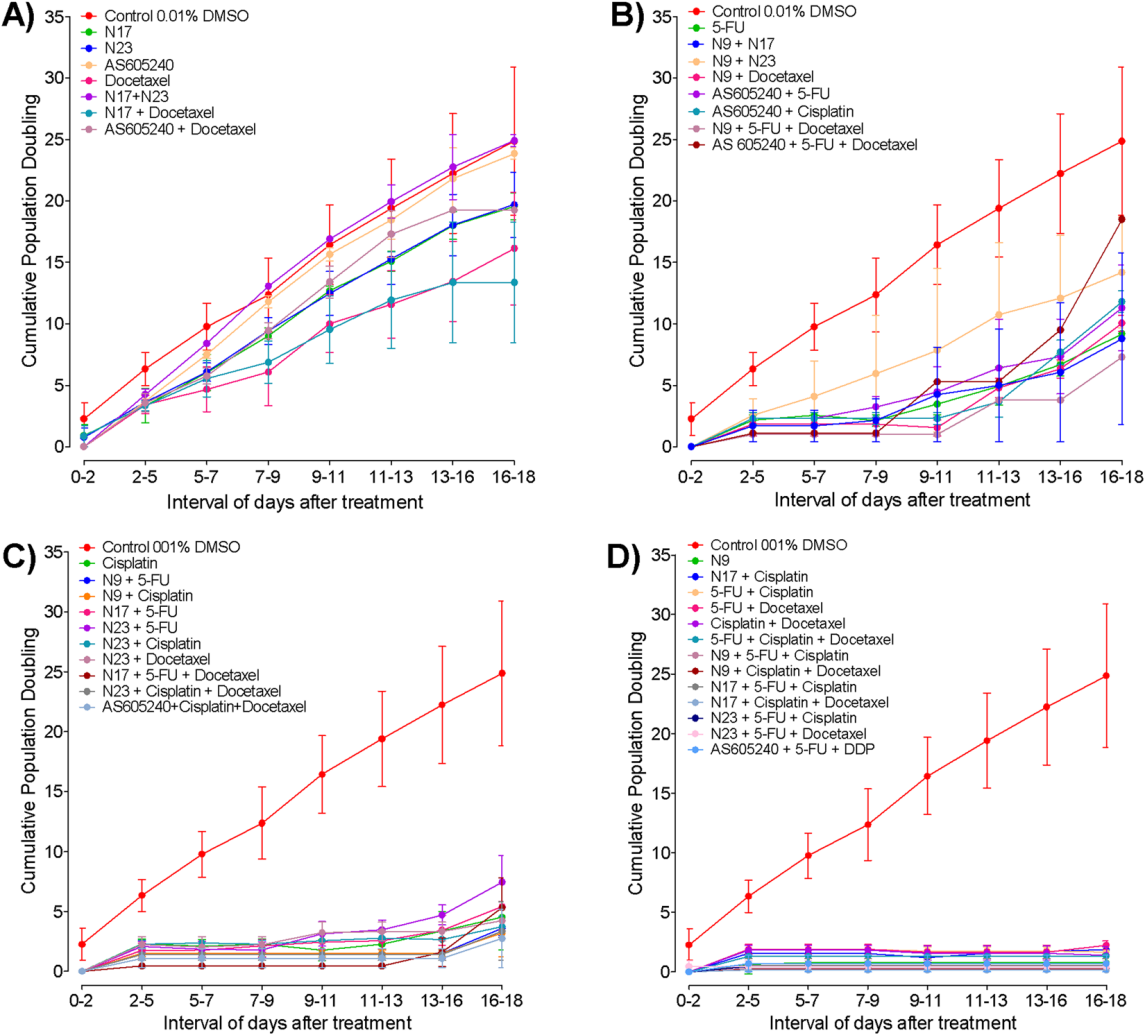
(**A**,**D**) Effects of quinoxalinic-derived chalcones, AS605240 and standard chemotherapies on cumulative population doubling (CPD) of OSCC cells. The HN30 cells were treated with N9 (chalcone N9; 15.61 µM), N17 (chalcone N17; 14.27 µM), N23 (chalcone N23; 17.22 µM), AS605240 (30 µM), 5FU (5-fluorouracil; 7.69 µM), cisplatin (10 µM), docetaxel (0.015 µM) alone or; N9 (7.81 µM), N17 (7.14 µM), N23 (8.61 µM), AS605240 (10 µM), 5FU (7.69 µM), cisplatin (10 µM), docetaxel (0.015 µM), incubated in combination schemes. (**A**) Represent the refractory treatments (growth between 2–9 days); (**B**) Early resistance profile (growth between 9–13 days); (**C**) Late resistance (growth between 13–18 days) and (**D**) Sensitive (growth only after 20 days). Each point represents the mean of at least 2 independent experiments performed in triplicate, and the lines indicate the standard error mean.

Recurrent head and neck cancer, including oral cancers, represent tumor deposits that arise locally or regionally after treatment. In those cases, prognosis is poor and the treatment options are very limited^26^. We carried out an assay to determine the cumulative population doubling (CPD), by testing the long-term antiproliferative effects of lead chalcones, AS605240 and the standard chemotherapy drugs, alone or in combination, for 18 days. A set of groups displayed a clear cell growth shortly after treatment, which remained increased throughout all the monitoring period, showing refractoriness to the treatments (Fig. 7A). In most cases, refractory features were seen in the isolated treatments, except by the combination of N17 plus N23, N17 and docetaxel or AS605240 plus docetaxel. This result indicates that these treatments may not be promising to treat OSCC.

Some treatments produced a growth profile that resemble an early resistance of OSCC tumor cells, with a proliferation ability recovery between 9 and 13 days after the treatment (Fig. 7B). The test groups showing this profile included 5-fluorouracil alone or combined with AS605240. Early resistance was also detected after incubation of AS605240 with cisplatin, or AS650240 plus docetaxel. Several combinations including N9, such as N9 plus N17 or N23, N9 plus docetaxel, or N9 plus 5-fluorouracil plus docetaxel were also associated with early resistance.

A third set of treatments led to late resistance, which was considered as the regrowth from days 13 to 18 onward (Fig. 7C). This outline was seen for cisplatin alone, and for most combinations including a lead chalcone and one reference drug. Late resistance was also observed for the triple association of N17 plus 5-fluorouracil and docetaxel; N23 plus cisplatin and docetaxel, or AS605240 with cisplatin and docetaxel.

Usually, the chemotherapy cycles are performed every 21 days in patients. In our protocol, some combinations inhibited the cell proliferation for up to 18 days, being defined as sensitive (Fig. 7D). Most of these treatments were composed of two standard chemotherapy agents, plus one chalcone (N9, 7.81 µM; N17, 7.14 µM; N23, 8.61 µM). Noteworthy, there was no growth retrieval, when HN30 cells were exposed to chalcone N9 alone (at 15.61 µM), what might be related to the marked antitumor effects of this quinoxalinic chalcone in the present study. Actually, N9 was the only single treatment that blocks cell regrowth at a long term. A similar response was achieved by the classic treatment regimen consisting of cisplatin plus 5-fluorouracil and docetaxel. This data reinforces the potential of N9 to the chemotherapy of OSCC, due to the ability to control cell growth for a prolonged period of time, minimizing the risk of recurrence.

Cancer chemotherapy generates several and important adverse events, mainly related to the effects on normal cells presenting high proliferation rates^26^. In this study, we provide preliminary results on the toxicity of quinoxaline-derived chalcones, by comparing their actions (alone or in combined schemes) in the MTT viability assay. A selectivity index (SI) was estimated on the basis of inhibition percentages obtained in non-tumor cells (HaCaT and Vero cells) versus tumor HN30 cells, as adapted from Tamokou *et al*.^27^. Data presented in the Supplementary Table S2 demonstrates SI values > 1 for different tested combinations in both HaCat and Vero cells, indicating that the reduction of the viability of the normal cells was below in relation to the tumor cells. Of note, this outcome was observed for the combination of cisplatin with each of the chalcones (N9, N17 and N23) or AS605240. Future studies using primary culture cells are still required to suitably determine the toxicity potential of the lead chalcones.

**Figure 8:**
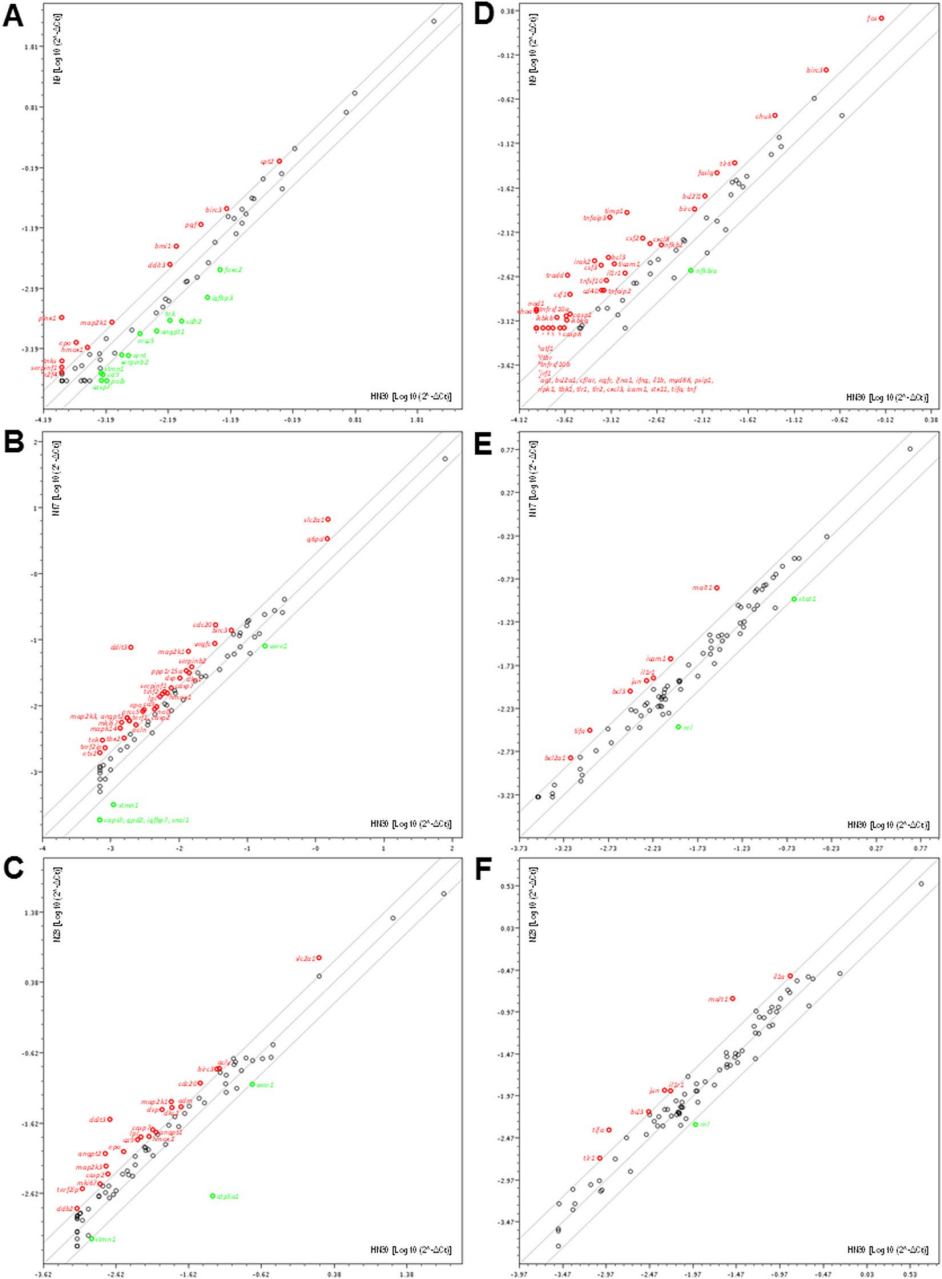
(**A**,**F**) Analysis of gene expression profile of quinoxalinic chalcones (N9, N17 and N23) using PCR array. Scatter plots show the up-regulation (red dots) or the down-regulation (green dots) of genes related to cancer-related pathways (A – chalcone N9; B – chalcone N17; C – chalcone N23) and NFκB-mediated signal transduction (D chalcone N9; E – chalcone N17; F – chalcone N23) using real-time PCR via *‘Human Cancer PathwayFinder PCR Array’ and ‘Human NFκB Signaling Pathway plus PCR Array’*. Changes in gene expression were calculated using the 2^-ΔCt method (RT^2^Profiler PCR array Data Analysis v3.5 - SABio/QIAGEN), and five stably expressed housekeeping genes (*B2M*, *Hprt1*, *Rpl13α*, *Gapdh*, and *β-actin*) were used for normalization of the results.

The progress of OSCC is related to gene deregulation and alterations of gene expression patterns^28^. The identification of these genes and pathways can point out new targets and treatment options for OSCC. To evaluate the effects of the selected quinoxaline-derived chalcones on the modulation of classical cancer pathways, we performed a PCR array comprising 84 genes. HN30 cells were treated with the lead compounds N9 (15.61 µM), N17 (14.27 µM) and N23 (17.22 µM), for 48 h, followed by the assessment of genes related to cell proliferation, apoptosis, cell cycle, angiogenesis, invasion and metastasis. It was possible to observe that the three chalcones modulated the expression of 51 different genes, from a total of 84 genes (Supplementary Table S3). N9 changed the expression of 22 genes; 10 of them were upregulated, and 12 were downregulated (Fig. 8A). N17 altered the expression of 37 genes, with an upregulation of 31 genes, and a downregulation of 6 genes (Fig. 8B). Finally, the chalcone N23 modulated 24 genes; 21 of them were upregulated, and only 3 genes were downregulated (Fig. 8C). The lead chalcones tested by us displayed distinct effects on the modulation of some cancer-related genes, what might explain, at least partly, the different profiles presented by the compounds on the functional assays of apoptosis and autophagy, besides the *CPD* protocol. For instance, N17 and N23 caused an upregulation of *ca9* and *casp7*, whereas the chalcone N9 produced a downregulation of these genes. The compound N9 also induced the downregulation of *angpt1*, *ercc5*, *serpinb2* and *tek*, whilst they were upregulated by N17 or N23, depending on the gene. The regulations of these genes can impact DNA repair pathways and angiogenesis^29–32^. The chalcone N9 caused a 2-fold increase of *ddit3* expression, while the chalcones N17 and N23 elicited 38- and 13-fold increases of this gene, respectively. *ddit3* plays an important role in cell stress responses, inducing apoptosis and cell cycle arrest^33^. The gene *atp5a1* had its expression reduced by 23-fold following the incubation of N23. Previous studies showed that ATP synthase α-subunit is overexpressed in liver, prostate, colon and breast cancers^34^. Furthermore, ATP synthase inhibitors reduce the proliferation of A549, MCF7 and MDA-MB-231 tumor cells, via the induction of apoptosis and cell cycle arrest, and by exerting anti-angiogenic actions^34,35^.

Noteworthy, 9 genes presented altered expression only after the treatment with compound N9. The downregulation of *arnt*, *cdh2*, *foxc2*, *polb*, and the overexpression of *e2f4* and pinx, can help to explain the expressive pro-apoptotic effects exhibited by N9 in our study. Indeed, these genes are able to modulate the survival rates of various tumor cell lines^36–40^. Most of them have also been involved in cell migration, invasiveness, metastasis and angiogenesis^37,39,41^, and their regulation likely contributes with the tumor control. Additionally, the downregulation of *arnt* and *foxc2* can reduce the proliferation of cells resistant to cisplatin and 5-FU^37,41^, corroborating the promising effects obtained by the combination of N9 with the reference chemotherapy drugs (Supplementary Table 1 and Fig. 7).

Previous studies demonstrated that a reduced expression of *cpt1* and *cpt2* genes contributed to a metabolic disruption and cachexia associated with several types of cancer^42^. The down-regulation of both genes has also been linked with the cytotoxic effects of some anticancer drugs, such as doxorubicin, cisplatin, carboplatin and oxaliplatin^42^. N9 upregulated the *cpt2* gene, what might represent an advantage for a novel anticancer candidate compound. Moreover, the chalcone N9 led to an upregulation of *bmi1*, and a downregulation of *igfbp3*. This result might elucidate the marked effects of the chalcone N9 in the CPD protocol (Fig. 7), considering that these genes are related to therapy failure and tumor relapse^43,44^, and that compound N9 alone prevented the cell proliferation for up to 20 days.

For validation of PCR array by real time quantitative RT-PCR, the gene *stmn1* was selected, by considering its similar regulation by the three chalcones. RT-PCR data confirmed that chalcones N9, N17 and N23 led to a downregulation of this gene in HN30 cells (Supplementary Table 4).

On the basis of the narrow relationship between cancer and inflammation^45^, we decided to evaluate the effects of the lead chalcones in the expression of genes implicated in inflammatory pathways. Thus, we employed the same methodology previously described in the evaluation of tumor gene modulation, by using the Human NF-κB Signaling Pathway plus PCR array kit, considering that NF-κB is the main transcription factor regulating the inflammatory protein expression. Chalcone N9 modulated a higher number of genes related to the inflammatory process than chalcones N17 and N23 (Supplementary Table S5). N9 upregulated 51 genes and downregulated only 1 gene, of the 84 genes analyzed (Fig. 8D). N17 altered the expression of 9 different genes; 7 of them were upregulated and 2 were downregulated (Fig. 8E). N23 modulated 8 genes, with an upregulation of 7 genes, and a downregulation of only 1 gene (Fig. 8F). The positive modulation of genes as *casp8*, *ifna1*, *ifng*, *irf1*, *rel*, *stxii*, *timp1*, *tnfrsf10a*, *tnfrsf10b* and *tnfsf10* could explain the ability of the tested chalcones to suppress the tumor cell proliferation and to induce apoptosis, especially the chalcone N9^46–52^. Besides, some of the evaluated genes (*agt*, *ripk1*, *tifa*, *tnfaip3* and *tradd*) might present dual effects on cancer, depending on the activated pathways and the tumor type^53–58^. Curiously, the chalcone N9 induced the upregulation of other genes of the TNF family, namely *cd40*, *fasg*, *ltbr*, *tnf* and *tnfaip2*, which might also display dual effects in the tumor cell death^58^. However, in the light of the available literature data, at this moment, we are not able to justify the anti-tumor effects displayed by N9 based on the upregulation of some inflammation-related genes, including *casp1*, *csf1*, *csf2*, *il1b*, *il1r*, *il1r1*, *myd88*, *nod1*, *tlr1*, *tlr2* and *tlr6*. Indeed, there is much to be investigated regarding the connection between these inflammatory molecules and cancer. The chalcones N17 and N23 led to the positive modulation of *casp2* and *casp7*, whereas the compound N9 increased the expression of casp1 and casp8, what might help to explain, at least partly, the distinct ability of the lead chalcones to modulate apoptosis, as demonstrated by different approaches in our study.

## Conclusion

Despite the advances in multimodality therapy, the overall survival of patients with OSCC remains poor and new treatment options are necessary. This study revealed that quinoxalinic-derived chalcones present marked *in vitro* anti-proliferative activities against OSCC. The cytotoxicity of these compounds on OSCC cells was mainly related to the induction of apoptosis and inhibition of Akt, besides the regulation of various genes related to tumorigenesis and inflammation. In parallel, cells that survived the acute stress caused by the chalcones triggered autophagy, probably as an escape mechanism to survive. The combination of the lead compounds with classical chemotherapy agents exhibited marked acute and long-term anti-tumor effects, with a good selectivity index. Intriguingly, the anti-tumor actions were prominently observed for the chalcones N9 and N17 that present two and three methoxy groups on the ring A, when compared to the subtle effects displayed by the monomethoxylated chalcone N23. It is possible to suggest that the quinoxalinic chalcones N17, and mainly N9, might represent promising compounds for the treatment of OSCC, although the *in vivo* efficacy of these compounds remains to be confirmed in future *in vivo* studies.

## Methods

### Chemistry

Twenty chalcones were prepared by aldolic condensation using quinoxalinic benzaldehyde 3 synthetized from 3,4-diaminobenzoic acid as presented in Supplementary Figure S11. The obtained yields were between 45% and 92%. The structures of the compounds were confirmed by chemical identification data: ^1^H NMR, ^13^C NMR and elemental analysis. ^1^H NMR spectra revealed that all structures were geometrically pure and configured E (*J*Hα-Hβ = 15.2–16.4 Hz).

#### Preparation of the compounds

The chalcones were prepared by aldolic condensation between quinoxalinic benzaldehyde and different acetophenones. The reagents used for the synthesis of the benzaldehyde, as a well as the acetophenone were obtained commercially (Merck, Sigma–Aldrich). The quinoxalinic benzaldehyde was obtained by condensation between 3,4-diaminob enzoic acid and glyoxal at reflux with ethanol and acetic acid, generating the acid derivated (**1**), as previously described by Melero-Pérez *et al*.^59^. Compound **1** was then reduced to its primary alcohol (**2**) with LiAlH_4_ after oxidized to aldehyde (**3**) with pyridine-chlorochromate in dichloromethane (Fig. 2). The chalcones (**4**–**12**) were prepared by magnetic agitation of acetophenone (2 mmol), methanol (30 mL), KOH 50% w/v (5 mL) and benzaldehyde **3** (2 mmol), at room temperature for 24 h. Distilled water and chloric acid 10% were added in the reaction for total precipitation of the compounds. The compounds were then obtained by vacuum filtration and later recrystallized in dichloromethane and hexane. The compounds N2 (R = 4′-OCH_3_), N3 (R = 3′,4′-diOCH_2_O), N4 (R = 4′-Br), N5 (R = H), N7 (R = 4′-NO_2_), N9 (R = 2′,5′-diOCH_3_), N10 (R = 3′,4′-diOCH_3_) and N12 (R = 2′,4′-diOCH_3_) were previously described by Mielcke *et al*. (2012)^34^. The compounds N15 (R = 3′,4′,5′-triOCH_3_), N16 (R = 3′-OCH_3_, 4′-OH), N17 (R = 2′,4′,5′-triOCH_3_), N18 (R = 3′,5′-diOCH_3_), N19 (R = 2-OCH_3_), N20 (R = 3′,5′-diOCH_3_, 4′-OH), N23 (R = 3′-OCH_3_) and N36 (R = 2′,3′,4′-triOCH_3_) were described by Winter *et al*. (2014)^36^. The compounds N24 (R = 4-imidazol), N33 (R = 4-CN), N34 (R = 3-CN) and N37 (R = 4-F) are new chalcones.

#### Physicochemical data of the compounds

The purified chalcones were obtained in yields between 45% and 92%. The structures were identified using melting points (m.p.), infrared spectroscopy (IR), 1 H and 13 C nuclear magnetic resonance spectroscopy (NMR), and for the unpublished ones, also elementary analyses. Melting points were determined with a Microquimica MGAPF-301 apparatus. NMR (1 H and 13 C NMR) were recorded on Varian Oxford AS-400 (400 MHz), using tetramethylsilane as an internal standard. Elementary analyses were obtained with a CHNS EA 1110. Percentages of C and H were in agreement with the product formula (within _0.4% of theoretical values to C). The purity of the synthesized chalcones was analyzed by thin-layer chromatography (TLC) using Merck silica pre-coated aluminum plates 200 mm in thickness with several solvent systems of different polarities. Compounds were visualized with ultraviolet light (254 and 360 nm) and using sulfuric anisaldehyde solution followed by heat as developing agent and purified by recrystallization from hexane and dichloromethane. 1 H NMR spectra revealed that all the structures were geometrically pure and configured E (JHα-Hβ = 15.2–16, 4 Hz).

### N24

(*E*)-1-(4-(1H-imidazol-1-yl)phenyl)-3-(quinoxalin-6-yl)prop-2-en-1-one. Yellow solid, m.p. 233-234 °C^1^H NMR (CDCl_3_) δ 7.27 (d, 1H, J = 8.0 Hz), 7,40 (d, 2H, J = 8.0 Hz), 7,46 (d, 1H, J = 8.0 Hz) 7.77 (d, 1H, J = 16.0 Hz), 7.87 (d, 2H, J = 8.0 Hz), 7.88 (d, 1H, J = 16.0 Hz), 8.03 (s, 1H), 8.04 (dd, 1H, J = 8.0/1.0 Hz), 8.12 (d, 1H, J = 8.0 Hz), 8.28 (s, 1H), 8.86 (dd, 2H, J = 8.0/1.0 Hz) ppm. ^13^C NMR (CDCl_3_) δ 116.75, 123.80, 124.24, 128.54, 129.75, 130.06, 130.71, 131.37, 133.56, 135.43, 136.20, 138.45, 140.32, 143.32, 145.08, 146.23, 147.08, 188.72 ppm. Anal. Calcd for C_20_H_14_N_4_O: C 73.61, H 4.32, N 17.17. Found: C 73.33, H 4.62, N 17.20. Yield: 77%.

### N33

(*E*)-4-(3-(quinoxalin-6-yl)acryloyl)benzonitrile. Yellow solid, m.p. 201-202 °C. ^1^H NMR (CDCl_3_) δ 8.80 (d, 2H, *J* = 8.0 Hz), 8.30 (s, 1H), 8.15 (d, 1H, *J* = 8.0 Hz), 8.08 (dd, 1H, *J* = 8.0 Hz), 8.00 (d, 1H, *J* = 16.0 Hz, Hα), 7.95 (d, 2H, *J* = 8.0 Hz), 7.70–7.68 (m, 2H), 7.69 (d, 1H, *J* = 16 Hz, Hβ) ppm. ^13^C NMR (CDCl_3_) δ 116.16, 123.45, 125.20, 126.15, 128.88, 130.25, 131.25, 132.72, 137.32, 142.59, 143.03, 143.11, 146.93, 147.14, 187.43 ppm. Anal. Calcd for C_18_H_11_N_3_O: C 75.78, H 3.89, N 14.73. Found: C 75.72, H 3.69, N 14.56. Yield: 78%.

### N34

(*E*)-3-(3-(quinoxalin-6-yl)acryloyl)benzonitrile. Yellow solid, m.p. 188-189 °C; ^1^H NMR (CDCl_3_) δ 7.16 (d, 1H, *J* = 8.0 Hz, H6′), 7.44 (t, 1H, *J* = 8.0 Hz, H5′), 7.48 (s, 1H, H2′), 7.65 (d, 1H, *J* = 8.0 Hz, H4′), 7.71 (d, 1H, *J* = 16.0 Hz, Hα), 8.00 (d, 1H, *J* = 16.0 Hz, Hβ), 8.05 (d, 1H, *J* = 8.0 Hz, H3), 8.15 (d, 1H, *J* = 8.0 Hz, H4), 8.30 (s, 1H, H1), 8.85 (d, 2H, *J* = 8.0 Hz, H6, H7) ppm; ^13^C NMR (CDCl_3_) δ 115.76, 119.61, 122.22, 124.49, 128.44, 129.70, 130.21, 130.52, 136.61, 139.19, 142.91, 143.13, 144.85, 145.08, 145.8, 188.74 ppm; Anal. Calcd for C_18_H_11_N_3_O: C 75.78, H 3.89, N 14.73. Found: C 75.72, H 3.69, N 14.56. Yield: 80%.

### N37

(*E)*-1-(4-fluorophenyl)-3-(quinoxalin-6-yl)prop-2-en-1-one. White solid, m.p. 189-190 °C. ^1^H NMR (CDCl_3_) δ 8.88 (d, 2H, *J* = 8.0 Hz), 8.37 (m, 3H), 8.20-8.16 (m, 3H), 8.03 (d, 1H, *J* = 16.0 Hz), 8.01 (m, 1H), 7.67 (d, 1H, *J* = 16 Hz) ppm. ^13^C NMR (CDCl_3_) δ 117.27, 124.24, 128.54, 129.75, 130.71, 131.37, 133.56, 136.20, 138.75, 142.60, 145.09, 146.23, 147.08, 169.05, 184.25 ppm. Anal. Calcd for C_17_H_11_FN_2_O: C 73.37, H 3.98, N 10.07. Found: C 73.52, H 3.99, N 10.23. Yield: 75%.

### Pharmacology

#### General cell culture procedures

HN30 (human oral squamous cell carcinoma) and HaCaT (immortalized human keratinocytes) cell lines were gently donated by Dr. Décio dos Santos Pinto Jr. (Oral Pathology Laboratory, FOUSP, Brazil). The Japanese Cancer Research Resources Bank (JCRB, Japan) supplied the SCC158 cell line (squamous cell carcinoma derived from the external acoustic meatus of the Fischer rat). Vero cells (epithelial cells from the African monkey kidney) were acquired from the Cell Bank of Rio de Janeiro (RJ, Brazil). The cells were grown in culture flasks with Dulbecco’s Modified Eagle’s medium (DMEM), supplemented with 10% (v/v) heat-inactivated fetal bovine serum (FBS), 0.1% amphotericin B and 1% penicillin/streptomycin. The cells were maintained at 37 °C, in a relative humidity of 95%, at a 5% CO_2_ atmosphere. All the reagents used for cell culture are from Gibco^TM^ (Thermo Fisher Scientific, Massachusetts, USA).

#### Cell viability assay

For screening purposes, the cell viability was evaluated by 3-(4,5-dimethylthiazol-2yl)-2,5-diphenyl tetrazolium bromide (MTT) assay. This assay is based on the ability of living cells to reduce the tetrazolium bromide salt (MTT [3-(4,5-dimethylthiazol-2-yl)-2,5-diphenyltetrazolium bromide]) in formazan crystals by the mitochondrial enzyme succinate dehydrogenase. SCC-158 and HN-30 cells were seeded in 96-well plates at the density of 1 × 10³ and 5 × 10³, respectively. After 24 h, the cells were exposed to increasing concentrations (0.29 µM to 38.42 µM) of twenty different quinoxaline-derived chalcones (Table 1) for 24 h, 48 h or 72 h. Parallel controls were carried out with DMEM plus 10% FBS (cell viability control) or 0.01% DMSO (vehicle control), in the absence of the test compounds. After the appropriate time intervals, the cells were incubated with 10% FBS-DMEM and MTT (5 mg/mL; Sigma-Aldrich, Missouri, USA) for 3 h. The supernatant was removed and 100 μg of dimethyl sulphoxide (DMSO; Sigma-Aldrich, Missouri, USA) was added to each well. The absorbance was read in a microplate reader at 490 nm (SpectraMax® M2e, with SoftMax® Pro Software, Molecular Devices, Pennsylvania, USA).

The effects of the selected compounds N9 (7.81 µM), N17 (7.14 µM), N23 (8.61 µM) were also tested in combination with standard chemotherapeutic drugs, currently used in clinics for treating OSCC, namely cisplatin (10 μM), 5-fluorouracil (7.69 μM) and docetaxel (0.015 μM). In this experimental set, the selective PI3Kγ inhibitor AS605240 (at 10 μM), used as the basis for synthesis of quinoxaline-derived chalcones, was also evaluated. The chalcones or the reference drugs were tested alone or in combination and the viability of the cell lines HN30, HaCat and Vero was analyzed by the MTT assay at 24 h, 48 h and 72 h.

For all experiments, at least three independent experiments (in triplicate) were performed. The results were expressed as the percentage of cell viability in relation to the negative control with 0.01% DMSO.

#### Clonogenic survival protocol

In this study, we assessed the number of cell colonies that were able to recover after the exposure to the selected chalcones. For this purpose, the growth and the proliferative characteristics of HN30 cells were determined after incubation with the selected quinoxaline-derived chalcones N9 (3.12 µM, 7.81 µM and 15.61 µM), N17 (2.85 µM, 7.14 µM and 14.27 µM) and N23 (3.44 µM, 8.61 µM and 17.22 µM) or with the positive control drug rapamycin (100 nM) and the starving in Hanks’ Balanced Salt solution (HBSS) for 48 h. After this period, the surviving cells were re-plated in six-well plates (100 cells/well), without treatments. After 7 days, the culture medium was changed. On the 12^th^ day after incubation, the cells were fixed with 4% paraformaldehyde, and stained with 0.5% crystal violet. The colonies with more than 50 cells were considered as survivors. They were identified under a microscope; photographs were taken for subsequent quantification of the number of colonies in the treatment and control groups. A minimum of three independent experiments (in duplicates) were carried out.

#### DAPI

DAPI is a fluorescent dye that preferentially stains dsDNA, allowing distinction of live cells and apoptotic cells. HN30 cells were seeded in 24-well plates at the density of 1 × 10^4^ cells/well. The following day, they were treated with the chalcones N9 (15.61 µM), N17 (14.27 µM) and N23 (17.22 µM). After 48 h, the cells were fixed with 4% paraformaldehyde, and stained with 300 nM of 4′,6-diamidino-2-phenylindole solution (DAPI; Sigma-Aldrich, Missouri, USA). The cells were photographed in a fluorescence microscope with appropriate filters (Olympus IX-71, Olympus Corporation, Tokyo, Japan), at 32X magnification. The images were analyzed using Image Pro-Plus 6.0 software (Media Cybernetics, Maryland, USA). To evaluate the nuclear morphometry and the nuclear irregularity index (NII), five parameters were used (area, roundness, aspect, radius ratio, and area box) and the nuclei were classified as irregular (I), large and regular (LR), large and irregular (LI), small and regular (SR), small (S), or small and irregular (SI) according the methodology by Fillipi-Chiela *et al*.^60^.

#### Characterization of cell death

To characterize the cell death process caused by the quinoxaline-related chalcones, HN30 cells were seeded on 24-well plates (3 × 10^4^ cells/well) and treated with the compounds N9 (15.61 µM), N17 (14.27 µM) and N23 (17.22 µM), for 18 and 24 h. The chemotherapy agent cisplatin (20 and 30 μM; 24 h) was used as a positive control. At the appropriate time-points, the cells were detached with 0.5% trypsin/EDTA solution (Gibco^TM^, Thermo Fisher Scientific, Massachusetts, USA). The cells were resuspended with annexin binding buffer, and incubated with 1 μL of annexin V-FITC plus 3 mM of propidium iodide (PI) (Dead Cell Apoptosis Kit with Annexin V FITC and PI, for flow cytometry, Molecular Probes^TM^, Thermo Fisher Scientific, Massachusetts, USA). Following incubation at 37 °C, for 15 minutes, the cells were analyzed by flow cytometry in Attune® Acoustic Focusing Cytometer (Applied Biosystems, Thermo Fisher Scientific, Massachusetts, USA). Data analysis was performed with the FlowJo Single Cell Analysis Software V10 (Tree Star Inc., California, USA).

#### Determination of kinase activity

This experimental set was conducted to verify the effects of the selected compounds on the activation of MAP kinases ERK 1/2 and p38, or the PI3K-related serine/threonine kinase AKT. For this purpose, HN30 cells (6 × 10^4^ cells/well) were plated in 6-well plates, and treated with the chalcones N9 (15.61 µM), N17 (14.27 µM) and N23 (17.22 µM) for 5, 15 or 30 min. Alternatively, the cells were treated at the same time intervals with the selective PI3Kγ inhibitor AS605240 (30 μM). The HN30 cells were detached with 0.5% trypsin/EDTA solution (GibcoTM, Thermo Fisher Scientific, Massachusetts, USA) and incubated with Phosflow Cytofix Buffer® at 37 °C, for 10 min. After washing, the cells were permeabilized with Phosflow Perm Buffer III® for 20 min, on ice. Subsequently, the antibodies anti–pp38, anti–pAKT and anti-pERK1/2 were incubated for 30 min in the dark, on ice. The analysis was made in an Attune® Acoustic Focusing Cytometer (Applied Biosystems, Thermo Fisher Scientific, Massachusetts, USA). Data was analyzed with FlowJo Single Cell Analysis Software V10 (Tree Star Inc., California, USA).

#### Acridine orange staining

Acridine orange (AO) is a fluorescent dye that stains (in red) the acidic vacuolar organelles (AVOs), mainly late autophagosomes/autolysosomes, in contrast with the green fluorescence from the whole cell. For the qualitative assay, HN30 cells were plated in 24-well plates (1 × 10^4^ cells/well), and treated with the chalcones N9 (15.61 µM), N17 (14.27 µM) and N23 (17.22 µM). After 48 h of treatment, the cells were incubated with AO (1 µg/mL; Sigma-Aldrich, Missouri, USA) for 15 min, and followed by visualization under a fluorescence microscope (Olympus IX-71, Olympus Corporation, Tokyo, Japan).

To quantify the percentage of cells with AVOs and the intensity of the red fluorescence, HN30 cells were plated in 24-well plates (3 × 10^4^ cells/well) and treated with the chalcones N9 (15.61 µM), N17 (14.27 µM) and N23 (17.22 µM), for 48 h. The cells were stained with AO (1.5 µg/mL), and analyzed in an Attune® Acoustic Focusing Cytometer (Applied Biosystems, Thermo Fisher Scientific, Massachusetts, USA). After 48 h, the cells were re-plated in a drug-free medium. The quantification of positive AVOs and red fluorescence was done at 72 and 120 h.

#### Bromodeoxyuridine (BrdU) incorporation and cell cycle determination

HN30 cells were seeded at 6 × 10^4^ cells/well in a 6-well plate and treated with the selected chalcones N9 (15.61 µM), N17 (14.27 µM) and N23 (17.22 µM), for 48 h. The cells were incubated with BrdU (10 µM) for 2 h (from 46 h to 48 h after treatment). Then, the cells were detached with 0.5% trypsin/EDTA solution (Gibco^TM^, Thermo Fisher Scientific, Massachusetts, USA), fixed with BD Cytofix® and permeabilized with BD Cytoperm Buffer®, followed by treatment with DNase and staining with fluorescent anti-BrdU and 7-AAD, according to the manufacturer’s protocol (FITC BrdU Flow Kit, BD Pharmingen^TM^, BD Biosciences, California, USA). Data was acquired using an Attune® Acoustic Focusing Cytometer (Applied Biosystems, Thermo Fisher Scientific, Massachusetts, USA), and analyzed with FlowJo Single Cell Analysis Software V10 (Tree Star Inc., California, USA). The cell cycle phase and DNA synthetic activity of cells was determined by the evaluation of total DNA expression (7-AAD) and incorporated BrdU levels (anti-BrdU FITC).

#### Cumulative population doubling

The long-term effects of a single treatment with the lead chalcones, alone or in combination schemes, were assessed by using the cumulative population doubling (CPD) assay. Briefly, HN30 cells were seeded in 24-well plates, at the density of 1 × 10^4^ cells/well. After 24 h, the cells were treated with the chalcones N9 (7.81 µM or 15.61 µM), N17 (7.14 µM or 14.27 µM) and N23 (8.61 µM or 17.22 µM), AS605240 (10 or 30 μM), cisplatin (10 μM), 5-fluorouracil (7.69 μM) and/or docetaxel (0.015 μM), alone or in combination. After 48 h, the cells were counted in an automated cell counter (Countess II FL, Life Technologies^TM^, Thermo Fisher Scientific, Massachusetts, USA), and re-plated at the same density, in drug-free medium. The counting was repeated every 2–4 days (according to the cell confluence), and the cells were re-plated at the same density for 18 days. The CPD was calculated using the equation PD = [log N(t) * logN(to)]/log 2, where N(t) is the number of cells/well at the time of the count, and N(to) is the initial number of cells. The sum of the PDs was plotted versus the time of the culture.

#### PCR array data analysis

The effects of the lead chalcones on several genes related to cancer and NF-κB-mediated cell signaling were assessed via PCR array. PCR array represents a method with high reproducibility, sensitivity, wide linear dynamic ranges, and specificity, similar to real-time PCR. For this experimental set, HN30 cells (1 × 10^6^ cells) were treated with the chalcones N9 (15.61 µM), N17 (14.27 µM) and N23 (17.22 µM), for 48 h. The total RNA was isolated with the RNeasy® mini kit (Qiagen®) and DNase digestion step was conducted with on-column DNase digestion step kit (Qiagen®), according to manufacturer’s recommendations. The yield and the quality of RNA samples was determined spectrophotometrically. One μg of total RNA sample was reverse transcribed using the First Strand cDNA kit RT2 (Qiagen®) in a final volume of 20 μL.

The kit Human Cancer PathwayFinder PCR array (PAHs-033Z; Qiagen®) was used to analyze the mRNA levels of 84 genes related to cell proliferation, apoptosis, cell cycle, angiogenesis, invasion and metastasis. The kit Human NF-κB Plus Signaling Pathway PCR array (PAHs-025Y; Qiagen®) was used to analyze mRNA levels of 84 genes related to signal transduction mediated NF-κB activation, according to the manufacturer’s instructions. Both kits are pre-designed cataloged pathway-focused PCR Arrays (Qiagen®). For data analysis, the 7500 Real-Time Systems Software v.2.0.6 was used (Applied Biosystems, Thermo Fisher Scientific, Massachusetts, USA).

The amplification protocol consisted of an initial denaturation at 95 °C for 10 min, followed by 40 cycles of denaturation at 95 °C for 15 s, renaturation and extension at 60 °C for 1 min. The expression of each gene was quantified based on Ct (threshold cycle), i.e. the number of cycles at which the fluorescence signal crosses the threshold. All of the genes represented by the array exhibited a single peak in the fusion curve, characteristic of this product. The analysis of gene expression was performed using the Excel-based PCR array Data Analysis Software (Qiagen®) (Qiagen, Hilden, Germany).

#### Validation of PCR array analysis by quantitative real-time RT-PCR

Quantitative real time PCR according to the method described by Sgnaolin *et al*.^61^. The gene *stmn1* was selected considering its similar regulation by the three chalcones. The primer was selected based on the publication by Zhang *et al*.^62^.

## Electronic supplementary material

Supplementary Information
