# Supplementary material for: Mechanisms underlying the antiproliferative effects of a series of quinoxaline-derived chalcones
Source: Sci Rep. 2017 Nov 20;7:15850. doi: 10.1038/s41598-017-16199-3 (PMC5696528; doi:10.1038/s41598-017-16199-3)
Supplement: Supplementary file 1 — Supplementary Information [file 41598_2017_16199_MOESM1_ESM.doc]

SUPPLEMENTARY MATERIAL

**Mechanisms underlying the antiproliferative effects of a series of quinoxaline-derived chalcones**

Tânia R. Mielcke^1,2^, Thaís C. Muradás^1,2^, Eduardo C. Filippi-Chiela^3^, Maria Eduarda A. Amaral^2,4^, Luiza W. Kist^1,5^, Maurício R. Bogo^1,4,5^, Alessandra Mascarello^6^, Patrícia D. Neuenfeldt^6^, Ricardo J. Nunes^6^, Maria M. Campos ^1,2,7^

¹ Postgraduate Program in Medicine and Health Sciences, PUCRS, Porto Alegre, RS, Brazil

^2^ Institute of Toxicology and Pharmacology, PUCRS, Porto Alegre, RS, Brazil

^3^ Postgraduate Program in Hepatology and Gastroenterology, UFRGS, Porto Alegre, RS, Brazil

^4^ Postgraduate Program in Cellular and Molecular Biology, PUCRS, Porto Alegre, RS, Brazil

^5^ Laboratory of Genomics and Molecular Biology, PUCRS, Porto Alegre, RS, Brazil

^6^ Department of Chemistry, UFSC, Florianópolis, SC, Brazil

^7^ School of Dentistry, PUCRS, Porto Alegre, RS, Brazil

**Supplementary Figure S1A-D.** **Effects of monomethoxylated quinoxalinic chalcones (chalcones N2, N16, N19 and N23) on the viability of HN-30 cells.** The cells were treated with concentrations ranging from 0.33 µM to 34.44 µM, for 24 (red line), 48 (blue line) and 72 h (green line). Each line represents the mean of at least three independent experiments performed in triplicate and the bars indicate the standard error mean. The dashed line represents 50 % of cell viability inhibition. Data were analyzed by one-way ANOVA, followed by Dunnett’s post-test. Significance between the treated groups and the control * (P < 0.05), ** (P < 0.01), *** (P <0.001).

**Supplementary Figure S2A-E.** **Effects of di-methoxylated quinoxalinic chalcones (chalcones N9, N10, N12, N18 and N20) on the viability of HN-30 cells.** The cells were treated with concentrations ranging from 0.30 µM to 31.22 µM, for 24 h (red line), 48 h (blue line) and 72 h (green line). Each line represents the mean of 3-4 independent experiments, performed in triplicate and the bars indicate the standard error mean. The dashed line represents 50 % of cell viability inhibition. Data were analyzed by one-way ANOVA, followed by Dunnett’s post-test. Significance between the treated groups and the control * (P < 0.05), ** (P < 0.01), *** (P <0.001).

**Supplementary Figure S3A-C.** **Effects of tri-methoxylated quinoxalinic chalcones (chalcones N15, N17 and N36) on the viability of HN-30 cells.** The cells were treated with the concentrations ranging from 0.29 µM to 28.54 µM, for 24 h (red line), 48 h (blue line) and 72 h (green line). Each line represents the mean of 3-4 independent experiments performed in triplicate and the bars indicate the standard error mean. The dashed line represents 50 % of cell viability inhibition. Data were analyzed by one-way ANOVA, followed by Dunnett’s post-test. Significance between the treated groups and the control * (P < 0.05), ** (P < 0.01), *** (P <0.001).

**Supplementary Figure S4A-H.** **Effects of non-methoxylated quinoxalinic chalcones (chalcones N3, N4, N5, N7, N24, N33, N34 and N37) on the viability of HN-30 cells.** The cells were treated with concentrations of ranging from 0.29 µM to 38.42 µM, for 24 h (red line), 48 h (blue line) and 72 h (green line). Each line represents the mean of 3-4 independent experiments performed in triplicate and the bars indicate the standard error mean. The dashed line represents 50 % of cell viability inhibition. Data were analyzed by one-way ANOVA, followed by Dunnett’s post-test. Significance between the treated groups and the control * (P < 0.05), ** (P < 0.01), *** (P <0.001).

**Supplementary Figure S5A-D.** **Effects of monomethoxylated quinoxalinic chalcones (chalcones N2, N16, N19 and N23) on the viability of SCC158 cells.** The cells were treated with concentrations ranging from 0.33 µM to 34.44 µM, for 24 h (red line), 48 h (blue line) and 72 h (green line). Each line represents the mean of 3-4 independent experiments, performed in triplicate and the bars indicate the standard error mean. The dashed line represents 50 % of cell viability inhibition. Data were analyzed by one-way ANOVA, followed by Dunnett’s post-test. Significance between the treated groups and the control * (P < 0.05), ** (P < 0.01), *** (P <0.001).

**Supplementary Figure S6A-E.** **Effects of di-methoxylated quinoxalinic chalcones (chalcones N9, N10, N12, N18 and N20) on the viability of SCC158 cells.** The cells were treated with concentrations ranging from 0.30 µM to 31.22 µM, for 24 h (red line), 48 h (blue line) and 72 h (green line). Each line represents the mean of 3-4 independent experiments performed in triplicate and the bars indicate the standard error mean. The dashed line represents 50 % of cell viability inhibition. Data were analyzed by one-way ANOVA, followed by Dunnett’s post-test. Significance between the treated groups and the control * (P < 0.05), ** (P < 0.01), *** (P <0.001).

**Supplementary Figure S7A-C.** **Effects of tri-methoxylated quinoxalinic chalcones (chalcones N15, N17 and N36) on the viability of SCC158 cells.** The cells were treated with concentrations ranging from 0.29 µM to 28.54 µM, for 24 h (red line), 48 h (blue line) and 72 h (green line). Each line represents the mean of at least 3-4 independent experiments performed in triplicate and the bars indicate the standard error mean. The dashed line represents 50 % of cell viability inhibition. Data were analyzed by one-way ANOVA, followed by Dunnett’s post-test. Significance between the treated groups and the control * (P < 0.05), ** (P < 0.01), *** (P <0.001).

**Supplementary Figure S8A-H.** **Effects of non-methoxylated quinoxalinic chalcones (chalcones N3, N4, N5, N7, N24, N33, N34 and N37) on the viability of SCC158 cells.** The cells were treated with concentrations ranging from 0.29 µM to 38.42 µM, for 24 h (red line), 48 h (blue line) and 72 h (green line). Each line represents the mean of 3-4 independent experiments performed in triplicate and the bars indicate the standard error mean. The dashed line represents 50 % of cell viability inhibition. Data were analyzed by one-way ANOVA, followed by Dunnett’s post-test. Significance between the treated groups and the control * (P < 0.05), ** (P < 0.01), *** (P <0.001).

**Supplementary Figure S9A-C.** **Analysis of the nuclear morphometry and the nuclear irregularity index (NII)**. HN30 cells were treated with the chalcones N9 (15.61 µM), N17 (14.27 µM) and N23 (17.22 µM) for 48 h and stained with DAPI. The cells were photographed in a fluorescence microscope, at 32X magnification. The data represents the distribution of nuclei in a plot of area versus NII (Nuclear Irregularity Index). N: Normal nuclei (nuclei used to establish the reference population. The ellipse represents the distribution for area and NII for normal nuclei), Ir: irregular, LR: Large Regular; LIr: Large Irregular; SR: Small and Regular; S: Small; SI: Small and Irregular – no data is represented, because any cell with these characteristics was observed.

**Supplementary Figure S10A-F. Effects of the selective PI3Kγ inhibitor AS605240 (30 μM) on the activation of MAP kinases ERK 1/2 and p38, or the PI3K-related serine/threonine kinase AKT.** HN30 cells were treated during 5, 15 or 30 min. (A-B) Histogram and bar chart showing the Akt phosphorylation. (C-D) Histogram and bar chart showing the Erk 1/2 phosphorylation. (E-F) Histogram and bar chart showing the p38 phosphorylation. The columns represent the mean of 3 independent experiments, and the lines indicate the standard error mean. Data were analyzed by two-way ANOVA, followed by Bonferroni’s post-test. Significantly different from the control group * (P < 0.05); ** (P < 0.01).

**Supplementary Figure S11. Synthetic route to obtain the twenty quinoxalinic-derived chalcones.** (i) glyoxal, acetic acid, CH3CH2OH, reﬂux; (ii) LiAlH4, THF; (iii) CCP, dichloromethane; (iv) corresponding acetophenones, methanol, 50% potassium hydroxide w/v, magnetic stirring, 24 h, room temperature.

**Supplementary Table S1. Effects of quinoxalinic-derived chalcones combined to reference chemotherapy agents on the viability of HN30 cells**

|  | **Inhibition percentages (mean ± SEM) at different time-points** | | |
| --- | --- | --- | --- |
| **Treatments** | **24 h** | **48 h** | **72 h** |
| **N9** | 22.6 ± 13.1 | 28.0 ± 18.2 | 31.8 ± 22.5 |
| **N17** | -0.2 ± 2.3 | 12.1 ± 1.6 | 8.3 ± 9.45 |
| **N23** | 14.7 ± 11.7 | 12.4 ± 6.1 | 18.7 ± 8.2 |
| **AS** | 6.5 ± 8.1 | -5.5 ± 9.5 | 10.0 ± 8.3 |
| **N9 + N17** | 36.3 ± 12.8 | 40.9 ± 17.2 | 51.5 ± 24.9 |
| **N9 + N23** | 38.2 ± 12.3 | 35.7 ± 20.0 | 38.5 ± 28.1 |
| **N17 + N23** | 18.7 ± 9.2 | 23.9 ± 16.3 | 19.6 ± 10.6 |
| **5FU** | 11.3 ± 4.9 | 45.1 ± 11.0 | 32.3 ± 20.1 |
| **DDP** | 10.9 ± 6.4 | 52.1 ± 12.9 | 59.3 ± 8.8 |
| **DCT** | 4.5 ± 12.6 | 33.5 ± 21.7 | 32.1 ± 14.7 |
| **N9 + 5FU** | 27.7 ± 10.5 | 39.0 ± 23.3 | 60.0 ± 29.8 |
| **N9 + DDP** | 36.7 ± 10.6 | 52.4 ± 24.6 | 66.0 ± 27.1 |
| **N9 + DCT** | 26.3 ± 2.1 | 29.7 ± 9.7 | 53.6 ± 0.28 |
| **N17 + 5FU** | 18.9 ± 4.2 | 41.7 ± 10.3 | 44.9 ± 14.8 |
| **N17 + DDP** | 28.5 ± 4.8 | 66.3 ± 4.3 | 66.3 ± 10.8 |
| **N17 + DCT** | 24.1 ± 15.1 | 6.2 ± 8.3 | 25.9 ± 12.8 |
| **N23 + 5FU** | 25.4 ± 5.0 | 35.3 ± 16.2 | 39.3 ± 16.8 |
| **N23 + DDP** | 40.4 ± 18.8 | 63.1 ± 7.3 | 52.0 ± 19.7 |
| **N23 + DCT** | 26.5 ± 19.9 | 45.9 ± 19.8 | 18.5 ± 3.0 |
| **AS + 5FU** | 25.7 ± 18.5 | 55.9 ± 7.7 | 40.6 ± 18.7 |
| **AS + DDP** | 36.2 ± 4.2 | 54.0 ± 21.0 | 46.0 ± 9.1 |
| **AS + DCT** | 47.4 ± 37.4 | 36.3 ± 17.0 | 6.1 ± 1.1 |
| **5FU + DDP** | 34.0 ± 9.5 | 69.1 ± 2.3 | 77.4 ± 5.0 |
| **5FU + DCT** | 21.0 ± 11.2 | 32.6 ± 5.4 | 32.6 ± 11.7 |
| **DDP + DCT** | 25.7 ± 14.1 | 49.4 ± 7.0 | 68.1 ± 4.5 |
| **DDP + 5FU + DCT** | 25.1 ± 17.9 | 62.0 ± 8.5 | 76.5 ± 6.2 |

N9 (chalcone N9; 7.81 µM), N17 (chalcone N17; 7.14 µM), N23 (chalcone N23; 8.61 µM), AS (AS605240; 10 µM), 5FU (5-fluorouracil; 7.69 µM), DDP (cisplatin;10 µM), DCT (docetaxel; 0.015 µM).

**Supplementary Table S2.** Selectivity index (SI) of quinoxalinic-derived chalcones combined to reference chemotherapy agents on the viability of HN30, HaCat and Vero cells.

|  | **Cell Ratio** | |
| --- | --- | --- |
| **Treatments** | **HaCat/HN30** | **Vero/HN30** |
| **Control** | 1.00 | 1.00 |
| **N9** | 0.77 | 0.80 |
| **N17** | 0.78 | 1.13 |
| **N23** | 0.92 | 0.93 |
| **AS** | 0.78 | 1.36 |
| **N9 + N17** | 1.19 | 0.96 |
| **N9 + N23** | 0.93 | 1.17 |
| **N17 + N23** | 0.68 | 1.12 |
| **5FU** | 1.03 | 1.79 |
| **DDP** | 0.99 | 2.06 |
| **DCT** | 1.02 | 1.91 |
| **N9 + 5FU** | 1.03 | 0.86 |
| **N9 + DDP** | 1.13 | 1.22 |
| **N9 + DCT** | 1.06 | 1.09 |
| **N17 + 5FU** | 1.01 | 1.48 |
| **N17 + DDP** | 1.24 | 2.41 |
| **N17 + DCT** | 0.53 | 1.08 |
| **N23 + 5FU** | 0.87 | 1.09 |
| **N23 + DDP** | 1.18 | 2.29 |
| **N23 + DCT** | 0.83 | 1.49 |
| **AS + 5FU** | 1.14 | 1.90 |
| **AS + DDP** | 1.02 | 2.13 |
| **AS + DCT** | 0.76 | 1.64 |
| **5FU + DDP** | 1.18 | 2.89 |
| **5FU + DCT** | 0.70 | 1.51 |
| **DDP + DCT** | 0.73 | 1.88 |
| **DCT + DDP + 5FU** | 0.90 | 2.27 |

N9 (chalcone 9; 7.81 µM), N17 (chalcone 17; 7.14 µM), N23 (chalcone 23; 8.61 µM), AS (AS605240; 10 µM), 5FU (5-fluorouracil; 7.69 µM), DDP (cisplatin; 10 µM), Docetaxel (0.015 µM). SI obtained from average Imax HaCat/Imax HN30 and Imax Vero/Imax HN30. Values represent the mean of at least three independent experiments in triplicate.

***Supplementary Table S3. Effects of quinoxalinic-derived chalcones on genes of cancer pathway on HN30 cells***

| **Gene ID** | **Gene Name** | **Fold change Chalcone 9** | **Fold change Chalcone 17** | **Fold change Chalcone 23** |
| --- | --- | --- | --- | --- |
| *ACLY* | ATP citrate lyase | -- | -- | 2.246 |
| *ADM* | Adrenomedullin | -- | -- | 2.1729 |
| *ANGPT1* | Angiopoietin 1 | -3.4121 | -- | 2.0499 |
| *ANGPT2* | Angiopoietin 2 | -- | 3.7157 | 5.129 |
| *ARNT* | Aryl hydrocarbon receptor nuclear translocator | -2.9911 | -- | -- |
| *ATP5A1* | ATP Synthase | -- | -- | -23.4774 |
| *BIRC3* | Baculoviral IAP Repeat Containing 3 | 2.3886 | 2.3487 | 2.3631 |
| *BMI1* | BMI1 polycomb ring finger oncogene | 3.6531 | -- | -- |
| *CA9* | Carbonic Anhydrase IX | -2.4155 | 2.0089 | 2.9605 |
| *CASP2* | Caspase-2 | -- | 3.2392 | 2.4109 |
| *CASP7* | Caspase-7 | -2.9184 | 2.4327 | 2.5165 |
| *CASP9* | Caspase-9 | -- | -3.694 | -- |
| *CDC20* | Cell Division Cycle 20 | -- | 4.7598 | 2.589 |
| *CDH2* | Cadherin 2, type 1, N-cadherin (neuronal) | -5.8088 | -- | -- |
| *CPT2* | Carnitine palmitoyltransferase 2 | 2.0699 | -- | -- |
| *DDB2* | Damage-Specific DNA Binding Protein 2 | -- | -- | 2.1301 |
| *DDIT3* | DNA-Damage-Inducible Transcript 3 | 2.3421 | 38.1834 | 13.7087 |
| *DKC1* | Dyskeratosis congenita 1 | -- | 2.2148 | 2.8145 |
| *DSP* | Desmoplakin | -- | 2.5749 | 3.7016 |
| *E2F4* | E2F transcription factor 4, p107/p130-binding | 2.0323 | -- | -- |
| *EPO* | Erythropoietin | 3.7491 | 2.8014 | 3.0613 |
| *ERCC5* | Excision Repair Cross-Complementation Group 5 | -2.0728 | 2.7723 | -- |
| *ETS2* | V-Ets Avian Erythroblastosis Virus E26 Oncogene Homolog 2 | -- | 2.7952 | -- |
| *FOXC2* | Forkhead box C2 (MFH-1, mesenchyme forkhead 1) | -3.3514 | -- | -- |
| *G6PD* | Glucose-6-phosphate dehydrogenase | -- | 2.2811 | -- |
| *GPD2* | Glycerol-3-phosphate dehydrogenase 2 | -- | -3.694 | -- |
| *HMOX1* | Heme Oxygenase 1 | 2.0392 | 2.2618 | 2.2714 |
| *IGFBP3* | Insulin-like growth factor binding protein 3 | -6.2048 | -- | -- |
| *IGFBP7* | Insulin-Like Growth Factor Binding Protein 7 | -- | -3.694 | -- |
| *LPL* | Lipoprotein Lipase | -- | 2.6433 | 2.8523 |
| *MAP2K1* | Dual specificity mitogen-activated protein kinase kinase 1 | 2.2067 | 4.7499 | 3.5425 |
| *MAP2K3* | Dual specificity mitogen-activated protein kinase kinase 3 | -- | 3.7376 | 3.2979 |
| *MAPK14* | Mitogen-activated protein kinase 14 | -- | 3.2816 | -- |
| *MKI67* | Marker Of Proliferation Ki-67 | -- | 3.8686 | 2.2931 |
| *NOL3* | Nucleolar Protein 3 | -- | 2.0317 | -- |
| *OCLN* | Occludin | -- | 2.1414 | -- |
| *PINX1* | PIN2/TERF1 interacting, telomerase inhibitor 1 | 16.6754 | -- | -- |
| *POLB* | Polymerase (DNA directed), beta | -3.4761 | -- | -- |
| *PPP1R15A* | Protein Phosphatase 1, Regulatory Subunit 15A | -- | 2.6228 | -- |
| *SERPINB2* | Serpin Peptidase Inhibitor, Clade B (Ovalbumin), Member 2 | -2.2748 | 2.4907 | -- |
| *SERPINF1* | Serpin Peptidase Inhibitor, Clade F (Alpha-2 Antiplasmin, Pigment Epithelium Derived Factor), Member 1 | 2.4318 | 2.6579 | -- |
| *SLC2A1* | Solute Carrier Family 2 (Facilitated Glucose Transporter) | -- | 4.3615 | 3.5783 |
| *SNAI1* | Snail Family Zinc Finger 1 | -- | -3.694 | -- |
| *STMN1* | Stathmin 1 | -2.2007 | -3.5357 | -2.0504 |
| *TBX2* | T-Box 2 | -- | 2.0528 | -- |
| *TEK* | TEK Tyrosine Kinase, Endothelial | -3.6898 | 4.0636 | -- |
| *TERF1* | Telomeric Repeat Binding Factor (NIMA-Interacting) 1 | -- | 3.1035 | -- |
| *TERF2I* | Telomeric Repeat Binding Factor 2 | -- | 2.7559 | 3.4337 |
| *TINF2* | TERF1 (TRF1)-Interacting Nuclear Factor 2 | -- | 2.6043 | -- |
| *VEGFC* | Vascular endothelial growth factor C | -- | 2.5921 | -- |
| *WEE1* | WEE1 G2 Checkpoint Kinase | -- | -2.3138 | -2.1301 |

***Supplementary Table S4. Validation of RT2 PCR array by real-time quantitative PCR for the gene stmn1***

|  | **Fold change Chalcone 9** | **Fold change Chalcone 17** | **Fold change Chalcone 23** |
| --- | --- | --- | --- |
| RT2 PCR array | -2.2 | -3.5 | -2.1 |
| Real-time quantitative PCR | -2.1 | -1.1 | -3.3 |

***Supplementary Table S5. Effects of quinoxalinic-derived chalcones on genes of the NFκB pathway on HN30 cells.***

| Gene ID | Gene Name | Fold change Chalcone 9 | Fold change Chalcone 17 | Fold change Chalcone 23 |
| --- | --- | --- | --- | --- |
| *AGT* | Angiotensinogen (serpin peptidase inhibitor, clade A, member 8) | 5.3155 | -- | -- |
| *ATF1* | Activating transcription factor 1 | 2.9320 | -- | -- |
| *BCL2A1* | BCL2-Related Protein A1 | 5.3155 | 2.1983 | -- |
| *BCL2L1* | BCL2-like 1 | 2.4053 | -- | -- |
| *BCL3* | B-Cell CLL/Lymphoma 3 | 5.4972 | 2.8434 | 2.0746 |
| *BIRC2* | Baculoviral IAP repeat containing 2 | 2.1969 | -- | -- |
| *BIRC3* | B-cell CLL/lymphoma 3 | 3.0309 | -- | -- |
| *CASP1* | Caspase 1, apoptosis-related cysteine peptidase (interleukin 1, beta, convertase) | 3.2974 | -- | -- |
| *CASP8* | Caspase 8, apoptosis-related cysteine peptidase | 2.6510 | -- | -- |
| *CD40* | CD40 molecule, TNF receptor superfamily member 5 | 2.7090 | -- | -- |
| *CFLAR* | CASP8 and FADD-like apoptosis regulator | 5.3155 | -- | -- |
| *CHUK* | Conserved helix-loop-helix ubiquitous kinase | 3.3384 | -- | -- |
| *CSF1* | Colony stimulating factor 1 (macrophage) | 5.5249 | -- | -- |
| *CSF2* | Colony stimulating factor 2 (granulocyte-macrophage) | 3.8155 | -- | -- |
| *CSF3* | Colony stimulating factor 3 (granulocyte) | 5.3823 | -- | -- |
| *CXCL3* | Chemokine (C-X-C motif) ligand 3 | 5.3155 | -- | -- |
| *CXCL8* | Interleukin 8 | 2.7799 |  |  |
| *EGFR* | Epidermal growth factor receptor | 5.3155 | -- | -- |
| *FASLG* | Fas ligand (TNF superfamily, member 6) | 3.2329 | -- | -- |
| *FOS* | FBJ murine osteosarcoma viral oncogene homolog | 2.8202 | -- | -- |
| *ICAM1* | Intercellular Adhesion Molecule 1 | 5.3155 | 2.3785 | -- |
| *IFNA1* | Interferon, alpha 1 | 5.3155 | -- | -- |
| *IFNG* | Interferon, gamma | 5.3155 | -- | -- |
| *IKBKB* | Inhibitor of kappa light polypeptide gene enhancer in B-cells, kinase beta | 4.2179 | -- | -- |
| *IKBKG* | Inhibitor of kappa light polypeptide gene enhancer in B-cells, kinase gamma | 3.0363 | -- | -- |
| *IL1A* | Interleukin 1, Alpha | -- | -- | 2.0279 |
| *IL1B* | Interleukin 1, beta | 5.3155 | -- | -- |
| *IL1R1* | Interleukin 1 Receptor, Type I | 2.3927 | 2.2298 | 2.0578 |
| *IRAK2* | Interleukin-1 receptor-associated kinase 2 | 7.1547 | -- | -- |
| *IRF1* | Interferon regulatory factor 1 | 4.4976 |  |  |
| *LTBR* | Lymphotoxin beta receptor (TNFR superfamily, member 3) | 3.4950 | -- | -- |
| *JUN* | Jun Proto-Oncogene | -- | 2.4691 | 2.4782 |
| *MALT1* | MALT1 Paracaspase | -- | 4.7813 | 5.0545 |
| *MYD88* | Myeloid differentiation primary response gene (88) | 5.3155 | -- | -- |
| *NFKB2* | Nuclear factor of kappa light polypeptide gene enhancer in B-cells 2 (p49/p100) | 2.0177 | -- | -- |
| *NFKBIA* | Nuclear factor of kappa light polypeptide gene enhancer in B-cells inhibitor, alpha | -2.0207 | -- | -- |
| *NOD1* | Nucleotide-binding oligomerization domain containing 1 | 8.6545 | -- | -- |
| *PSIP1* | PC4 and SFRS1 interacting protein 1 | 5.3155 | -- | -- |
| *REL* | V-Rel Avian Reticuloendotheliosis Viral Oncogene Homolog | -- | -3.1778 | -2.3654 |
| *RHOA* | Ras homolog gene family, member A | 8.3558 | -- | -- |
| *RIPK1* | Receptor (TNFRSF)-interacting serine-threonine kinase 1 | 5.3155 | -- | -- |
| *STAT1* | Signal Transducer And Activator Of Transcription 1 | -- | -2.1292 | -- |
| *STX11* | Syntaxin 11 | 5.3155 | -- | -- |
| *TBK1* | TANK-binding kinase 1 | 5.3155 | -- | -- |
| *TICAM1* | Toll-like receptor adaptor molecule 1 | 4.0011 | -- | -- |
| *TIFA* | TRAF-Interacting Protein With Forkhead-Associated Domain | 5.3155 | 2.8589 | 3.6381 |
| *TIMP1* | TIMP metallopeptidase inhibitor 1 | 11.1198 | -- | -- |
| *TLR1* | Toll-Like Receptor 1 | 5,3155 | -- | 2.1464 |
| *TLR2* | Toll-like receptor 2 | 5.3155 | -- | -- |
| *TLR6* | Toll-like receptor 6 | 2.6926 | -- | -- |
| *TNF* | Tumor necrosis factor | 5.3155 | -- | -- |
| *TNFAIP2* | Tumor necrosis factor, alpha-induced protein 2 | 2.6199 | -- | -- |
| *TNFAIP3* | Tumor necrosis factor, alpha-induced protein 3 | 15.0451 | -- | -- |
| *TNFRSF10A* | Tumor necrosis factor receptor superfamily, member 10a | 3.4404 | -- | -- |
| *TNFRSF10B* | Tumor necrosis factor receptor superfamily, member 10b | 3.9137 | -- | -- |
| *TNFSF10* | Tumor necrosis factor (ligand) superfamily, member 10 | 3.1612 | -- | -- |
| *TRADD* | TNFRSF1A-associated via death domain | 9.6750 | -- | -- |
